# Supplementary material for: GeneGini: Assessment via the Gini Coefficient of Reference “Housekeeping” Genes and Diverse Human Transporter Expression Profiles
Source: Cell Syst. 2018 Feb 28;6(2):230–244.e1. doi: 10.1016/j.cels.2018.01.003 (PMC5840522; doi:10.1016/j.cels.2018.01.003)
Supplement: Document S1. Figures S1–S8 and Tables S3 and S4 [file mmc1.pdf]

Cell Systems, Volume *6*

Supplemental Information

GeneGini: Assessment via the Gini Coefficient

of Reference "Housekeeping" Genes

and Diverse Human Transporter Expression Profiles

Steve O'Hagan, Marina Wright Muelas, Philip J. Day, Emma Lundberg, and Douglas B. Kell

SUPPLEMENTARY INFORMATION

The extra subsetted data that we give in the Supplementary Information are as follows:

Supplementary Table S1. Related to Fig 1. Expression profiles of the SLC transporters. Separate XL File: Supplementary Table S1 SLC_transporters_RNA_data_HPA.xlsx

Supplementary Table S2. Related to Fig 4. Expression profiles of the ABC transporters. Separate XL File: Supplementary Table S2 ABC_transporters_RNA_data_HPA.xlsx

Supplementary Table S3. Related to STAR Methods. A previously proposed set of useful reference genes, annotated here with their correct names and Uniprot IDs, together with their median expression levels and Gini indices in tissues as determined in this work.

Supplementary Table S4. Related to STAR Methods. Some genes that have previously been proposed as housekeeping or reference genes.

Supplementary Figures.

S1 (Relates to Fig 1.) Expression profiling of various SLC transporters in 59 tissues. Minimum and maximum expression levels of various SLCs in the 59 tissues considered (those with undetectable expression (i.e. <0.01 TPM, coded as zero) are not shown).

S2. (Relates to Fig 1.) Expression profiling of various SLC transporters in 59 tissues. The expression level of SLC35A4 is relatively homogeneous, with ¾ of all tissues within a factor two.

S3. (Relates to Fig 1.) Expression profiling of various SLC transporters in 59 tissues. The expression levels of SLC35F2 vary much more considerably, by a range of ~200 in these 59 tissue types.

S4. (Relates to Fig 1.) Expression profiling of various SLC transporters in 59 tissues. Expression profile of the transcripts for SLC22A4.

S5. (Relates to Fig 2.) Zoomed-in version of cluster 1 of Figure 2.

S6. (Relates to Fig 2.) Zoomed-in version of cluster 2 of Figure 2.

S7. (Relates to Fig 2.) Zoomed-in version of cluster 3 of Figure 2.

S8. (Relates to Fig 2.) Zoomed-in version of cluster 4 of Figure 2.

**Gene name**	**Protein name**	**Uniprot ID**	**Gini index in tissues**	**Median expression level (TPM)**
C1orf43	Chromosome 1 open reading frame 43	Q9BWL3	0.204	137
CHMP2A	Charged multivesicular body protein 2A	O43633	0.141	126
EMC7	ER membrane protein complex subunit 7	Q9NPA0	0.210	69
GPI	Glucose-6-phosphate isomerase	P06744	0.259	137
PSMB2	Proteome subunit beta type 2	P49721	0.186	32
PSMB4	Proteome subunit beta type 4	P28070	0.200	209
RAB7A	RAS-related protein 7A	P51149	0.171	167
REEP5	Receptor expression-enhancing protein 5	Q00765	0.315	65
SNRPD3	Small nuclear ribonucleoprotein Sm D3	P62318	0.192	55
VCP	Transitional endoplasmic reticulum ATPase (originally valosin containing protein)	P55072	0.198	48
VPS29	Vacuolar protein sorting associated protein 29	Q9UBQ0	0.146	74

Supplementary Table S3. A previously proposed set of useful reference genes, annotated here with their correct names and Uniprot IDs, together with their median expression levels and Gini indices in tissues as determined in this work.

Gene	Protein	Uniprot ID	Gini index
GAPDH	Glyceraldehyde 3-phosphate dehydrogenase	P04406	0.344
LDHA	Lactate dehydrogenase subunit A	P00338	0.32
SDHA	Succinate dehydrogenase subunit A	P31040	0.308
HRPT1	Hypoxanthine phosphoribosyl transferase 1	P00492	0.277
HBS1L	*HBS1*-like protein	Q9Y450	0.184
OAZ1	Ornithine decarboxylase antizyme 1	P54368	0.202
PPIA1	Peptidyl-prolyl cis-trans isomerase	P62937	0.24
AHSP	Alpha-haemoglobin stabilising protein	Q9NZD4	0.97
B2M	β_2-microglobulin	P61769	0.349
ACTB	β-actin	P60709	0.291
HMBS	Porphobilinogen deaminase	P08397	0.303
UBC	Polyubiquitin C	P0CG48	0.183
POLR2F	DNA-directed RNA polymerases I, II, and III subunit RPABC2	P61218	0.235
GUSB	β-glucuronidase	P08236	0.25
TBP	TATA-box binding protein	P20226	0.22
YWHAZ	14-3-3 protein zeta/delta	P63104	0.255

Supplementary Table S4. Some genes that have previously been proposed as housekeeping or reference genes.

S1

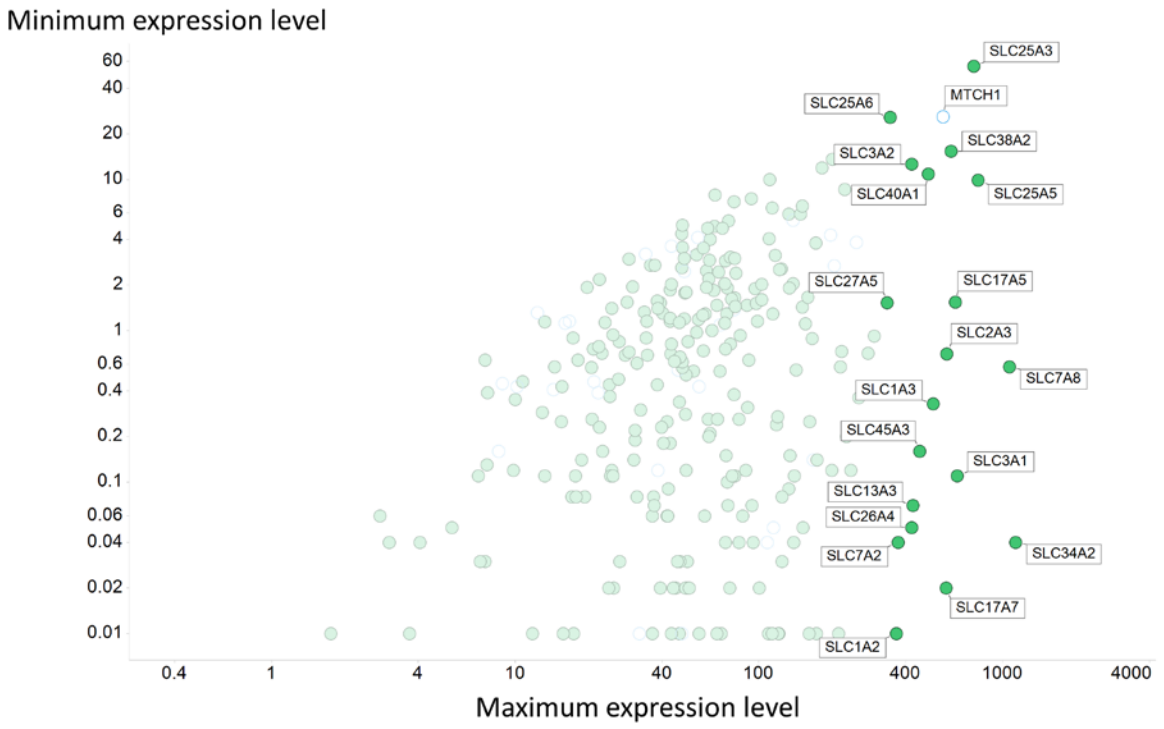

Supplementary Fig S1 (Relates to Fig 1.) Expression profiling of various SLC transporters in 59 tissues. Minimum and maximum expression levels of various SLCs in the 59 tissues considered (those with undetectable expression (i.e. <0.01 TPM, coded as zero) are not shown).

S2

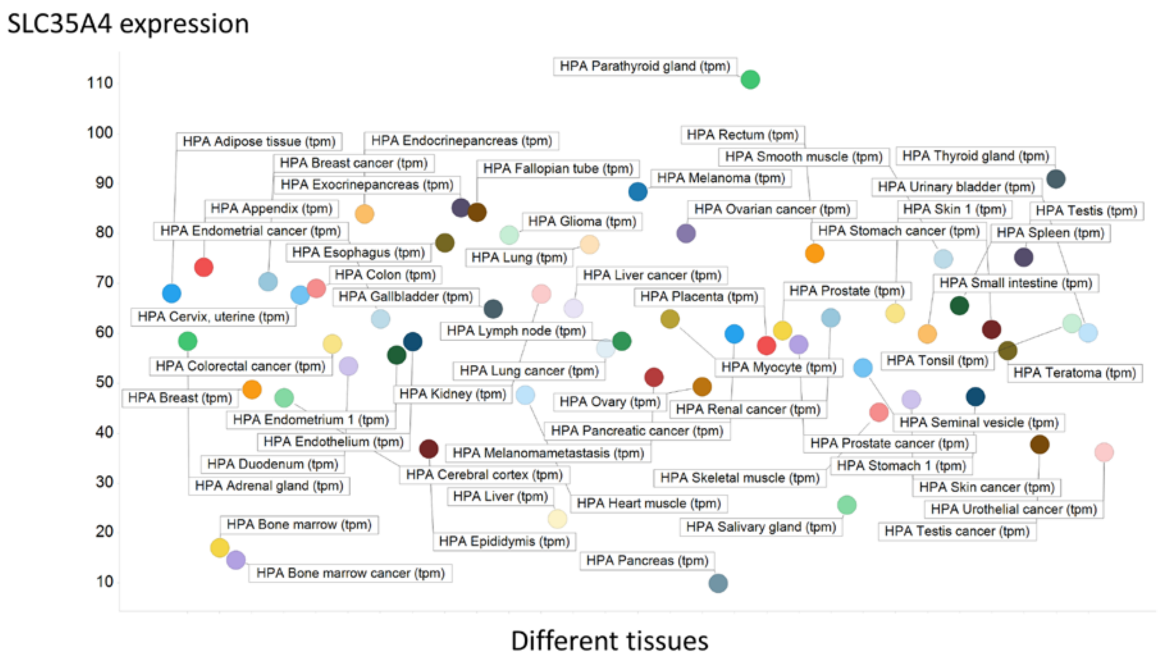

Supplementary Fig S2. (Relates to Fig 1.) Expression profiling of various SLC transporters in 59 tissues. The expression level of SLC35A4 is relatively homogeneous, with ¾ of all tissues within a factor two.

S3

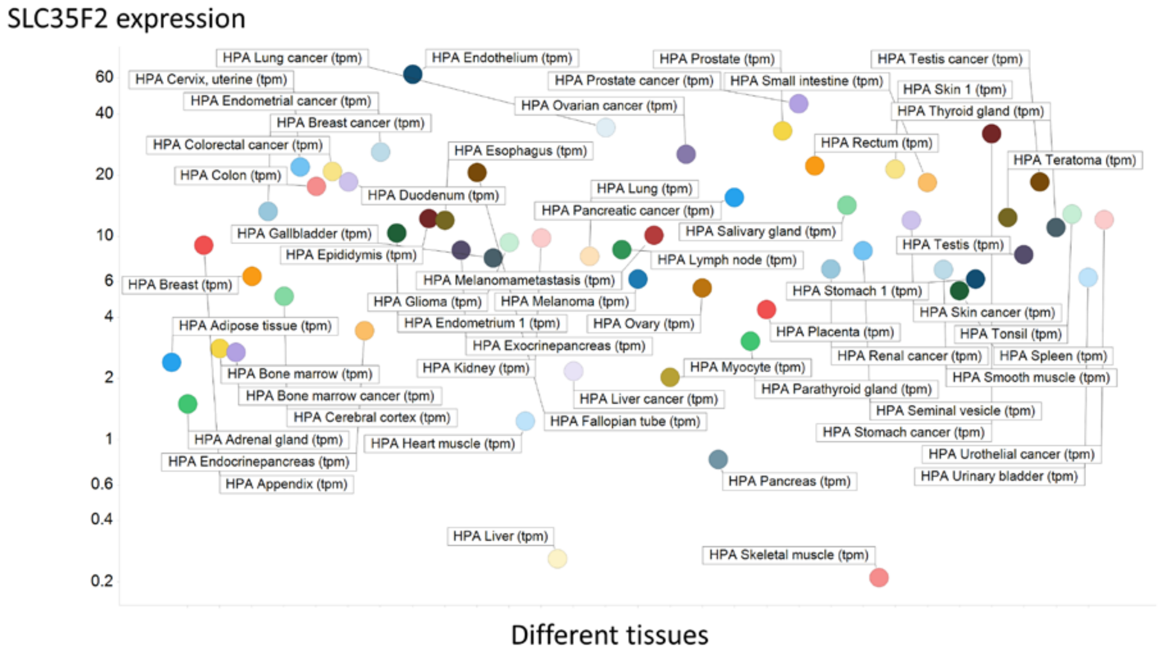

Supplementary Fig S3. (Relates to Fig 1.) Expression profiling of various SLC transporters in 59 tissues. The expression levels of SLC35F2 vary much more considerably, by a range of ~200 in these 59 tissue types.

S4

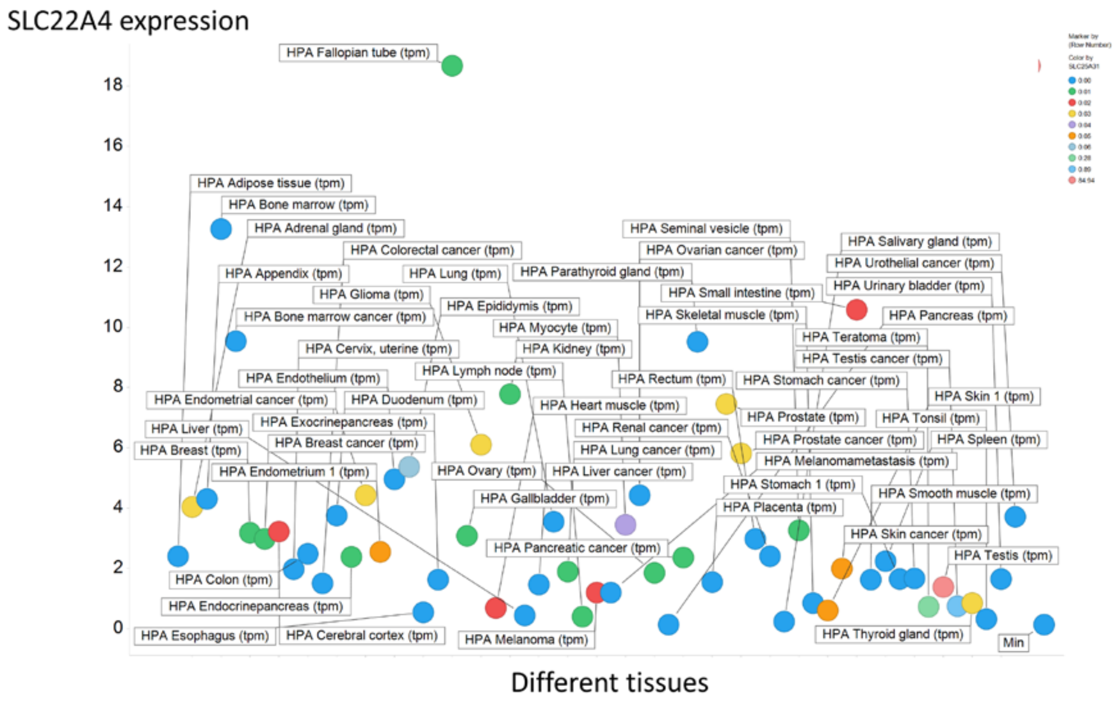

Supplementary Fig S4. (Relates to Fig 1.) Expression profiling of various SLC transporters in 59 tissues. Expression profile of the transcripts for SLC22A4.

S5

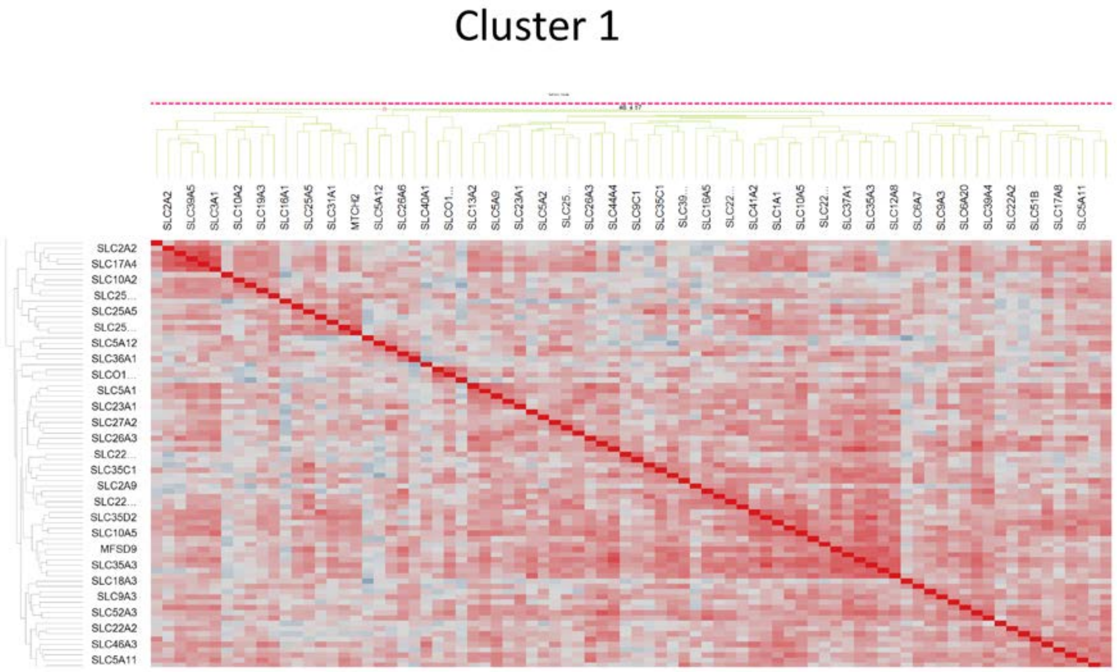

Supplementary Fig S5. (Relates to Fig 2.) Zoomed-in version of cluster 1 of Figure 2.

S6

Cluster 2

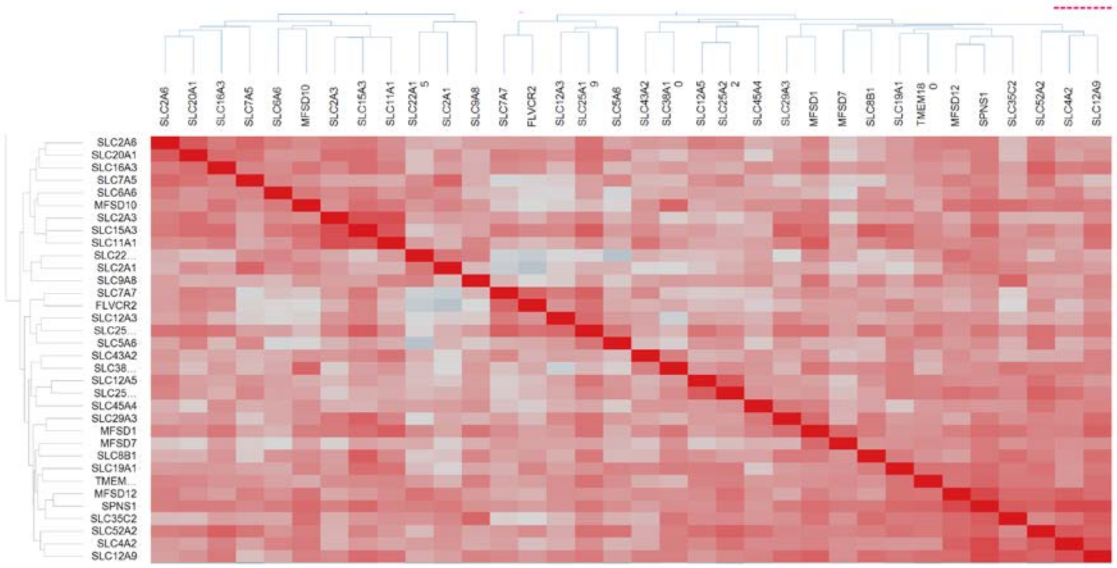

Supplementary Fig S6. (Relates to Fig 2.) Zoomed-in versions cluster2 of Figure 2.

S7

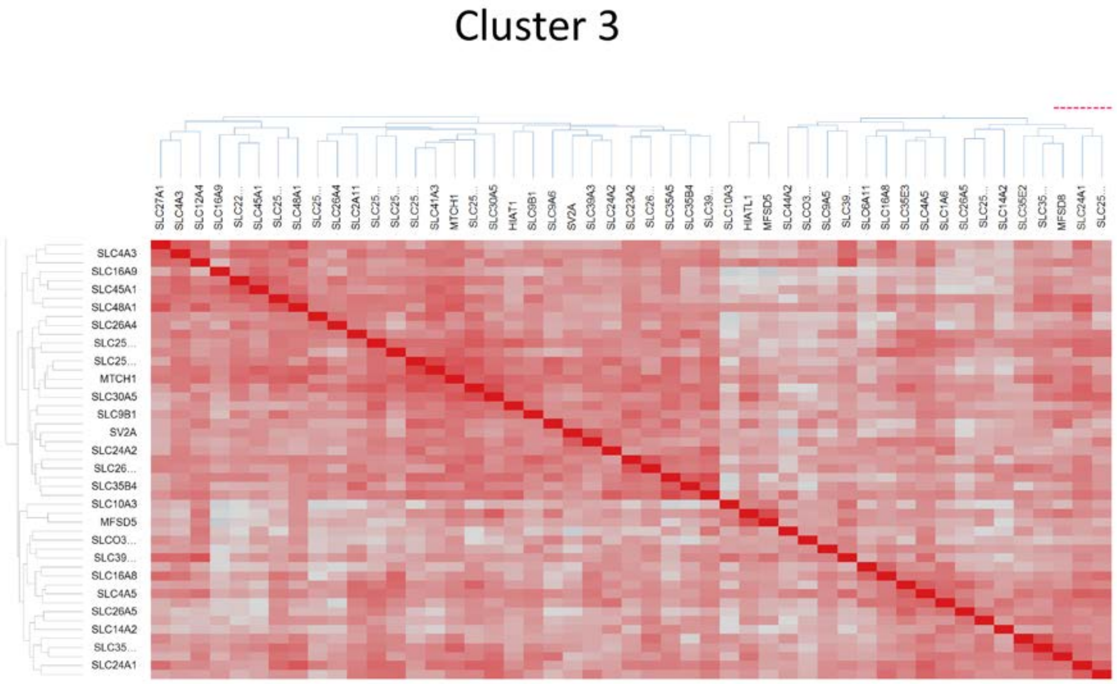

Supplementary Fig S7. (Relates to Fig 2.) Zoomed-in version cluster 3 of Figure 2.

S8

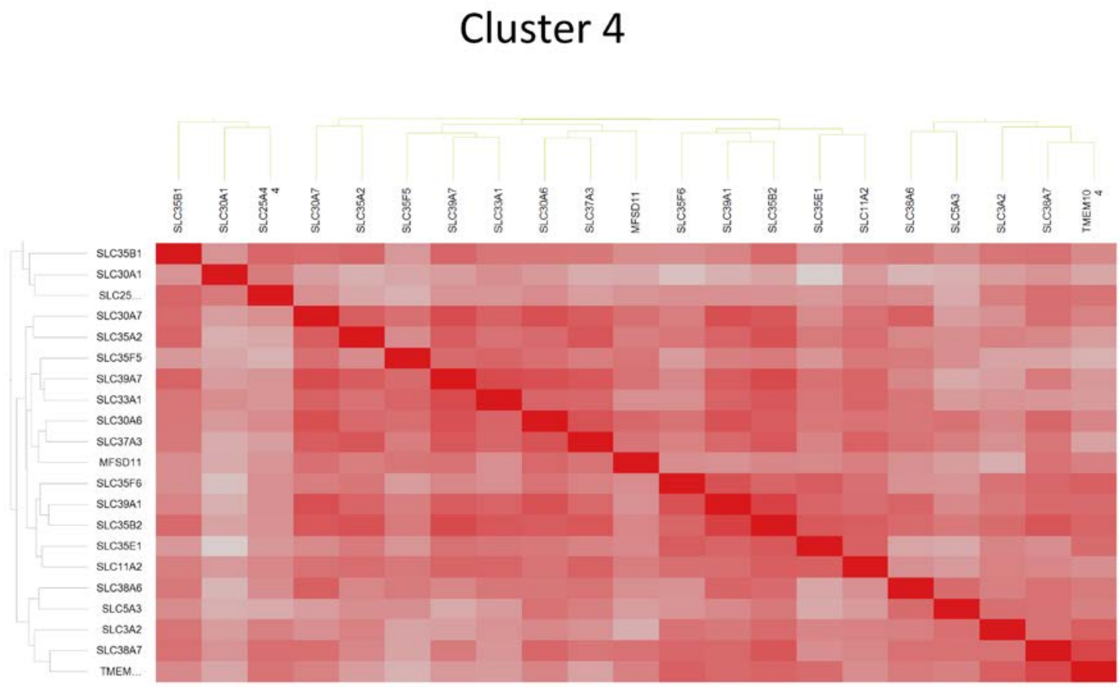

Supplementary Fig S8. (Relates to Fig 2.) Zoomed-in version of cluster 4 of Figure 2.
