# Supplementary material for: GeneGini: Assessment via the Gini Coefficient of Reference “Housekeeping” Genes and Diverse Human Transporter Expression Profiles
Source: Cell Syst. 2018 Feb 28;6(2):230–244.e1. doi: 10.1016/j.cels.2018.01.003 (PMC5840522; doi:10.1016/j.cels.2018.01.003)
Supplement: Document S2. Article plus Supplemental Information [file mmc4.pdf]

Cell Systems

Article

GeneGini: Assessment via the Gini Coefficient of Reference “Housekeeping” Genes and Diverse Human Transporter Expression Profiles

Graphical Abstract

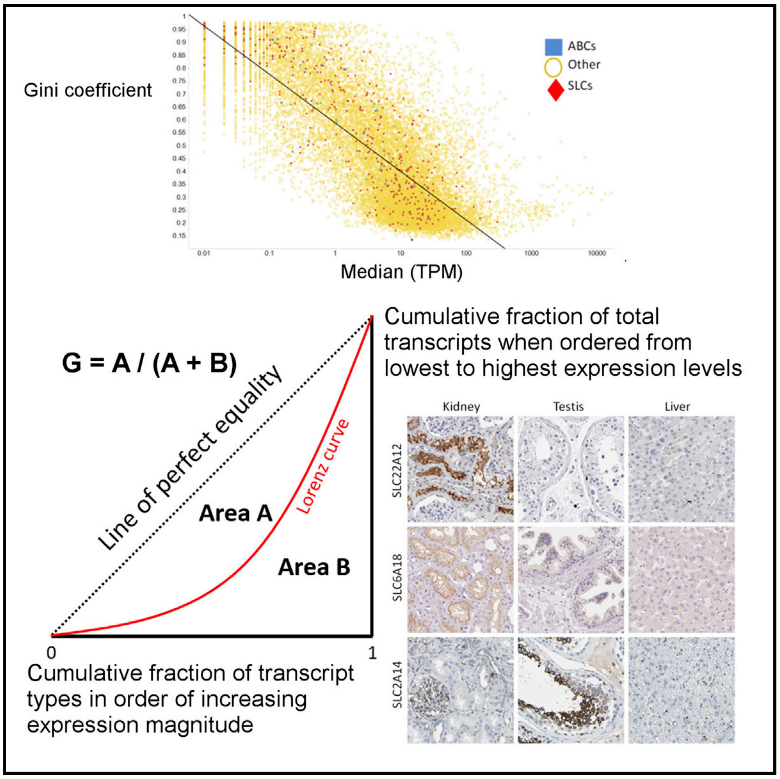

Highlights

- Gini index (0–1) is a convenient means of summarizing inequalities of distribution
- We apply it to two, large transcriptome datasets from tissues and cell lines
- Membrane transporters (SLCs) have unusually heterogeneous distributions
- Low Gini index transcripts make great reference genes; we describe many new ones

Authors

Steve O’Hagan, Marina Wright Muelas, Philip J. Day, Emma Lundberg, Douglas B. Kell

Correspondence

emma.lundberg@scilifelab.se (E.L.), dbk@manchester.ac.uk (D.B.K.)

In Brief

The Gini index (coefficient) is used by economists to describe inequalities in wealth distribution in populations and varies between 0 (full equality) and 1 (extreme inequality). We here adopt it to describe, in a simple way, the distributions of expression levels of different genes between tissues or cell lines. We find that uptake (SLC) and efflux (ABC) transporters are more heterogeneously distributed than are members of most other gene families. By contrast, genes with a low Gini coefficient must be stably expressed and can be proposed as reference genes for normalization in expression profiling studies. As judged by this criterion, many previously unidentified reference genes may be proposed.

O’Hagan et al., 2018, Cell Systems *6*, 230–244
February 28, 2018 © 2018 The Author(s). Published by Elsevier Inc.
https://doi.org/10.1016/j.cels.2018.01.003

OPEN ACCESS
CellPress

Cell Systems
Article

GeneGini: Assessment via the Gini Coefficient of Reference "Housekeeping" Genes and Diverse Human Transporter Expression Profiles

Steve O'Hagan,[1,2] Marina Wright Muelas,[1,2] Philip J. Day,[2,3] Emma Lundberg,[4,*] and Douglas B. Kell[1,2,5,*]
[1]School of Chemistry
[2]The Manchester Institute of Biotechnology
131, Princess Street, Manchester M1 7DN, UK
[3]Faculty of Biology, Medicine and Health, The University of Manchester, Oxford Road, Manchester M13 9PL, UK
[4]Science for Life Laboratory, Royal Institute of Technology (KTH), SE-17121 Solna, Sweden
[5]Lead Contact
*Correspondence: emma.lundberg@scilifelab.se (E.L.), dbk@manchester.ac.uk (D.B.K.)
https://doi.org/10.1016/j.cels.2018.01.003

SUMMARY

The expression levels of SLC or ABC membrane transporter transcripts typically differ 100- to 10,000-fold between different tissues. The Gini coefficient characterizes such inequalities and here is used to describe the distribution of the expression of each transporter among different human tissues and cell lines. Many transporters exhibit extremely high Gini coefficients even for common substrates, indicating considerable specialization consistent with divergent evolution. The expression profiles of SLC transporters in different cell lines behave similarly, although Gini coefficients for ABC transporters tend to be larger in cell lines than in tissues, implying selection. Transporter genes are significantly more heterogeneously expressed than the members of most non-transporter gene classes. Transcripts with the stablest expression have a low Gini index and often differ significantly from the "housekeeping" genes commonly used for normalization in transcriptomics/qPCR studies. PCBP1 has a low Gini coefficient, is reasonably expressed, and is an excellent novel reference gene. The approach, referred to as GeneGini, provides rapid and simple characterization of expression-profile distributions and improved normalization of genome-wide expression-profiling data.

INTRODUCTION

Given that the basic genome of a differentiated organism is constant between cells (and we here ignore epigenomics), what mainly discriminates one cell type from another is its expression profile. The "surfaceome" (those proteins expressed on the cell surface) attracts our interest in particular, as it contains the transporters that determine which nutrients (and xenobiotics such as drugs) are taken up by specific cells (da Cunha et al., 2009; Palm and Thompson, 2017). Transporters are the second largest component of the membrane proteome (Almén et al., 2009), and also a (surprisingly) understudied clade (César-Razquin et al., 2015). They are classified into solute carriers (SLCs) (Colas et al., 2016; Fredriksson et al., 2008; Hediger et al., 2013; Perland and Fredriksson, 2017; Schlessinger et al., 2010; Sreedharan et al., 2011), mainly involved in uptake, and ABC transporters (ABCs), mainly involved in efflux (e.g., Chen et al., 2016; Eadie et al., 2014; Montanari and Ecker, 2015; Rees et al., 2009).

Transporters are also responsible for the uptake of pharmaceutical drugs and xenobiotics into cells, and their efflux therefrom (Colas et al., 2016; Dobson and Kell, 2008; Giacomini and Huang, 2013; Giacomini et al., 2010; Kell, 2015, 2016; Kell et al., 2011, 2013; Kell and Oliver, 2014; Lin et al., 2015; Stanley et al., 2009). This means that, to understand drug distributions, we must understand transporter distributions. In many cases, we do not know either the "natural" (O'Hagan and Kell, 2017b, 2018; Perland and Fredriksson, 2017) or the pharmaceutical drug substrates of these transporters, and one clue to this may be to understand transporters' differential tissue distribution.

In the present work we used absolute transcription profiles acquired (via RNA sequencing) as part of the tissue atlas (Uhlén et al., 2015) and cell atlas (Thul et al., 2017). Altogether there are four main datasets, namely 409 SLCs in 59 tissue types and 56 cell lines, and 48 ABCs in the same tissue types and cell lines. Some of the SLCs do not (yet) have the official terminology (Perland and Fredriksson, 2017; Sreedharan et al., 2011), but, based on a variety of phylogenetic and other evidence, as well as their UniProt annotations, they clearly have this function, and these are noted accordingly. Similarly, some of the "ABC" families (especially family F) are probably not functionally membrane transporters, but they are nonetheless included.

The availability of extensive and high-quality transcriptomic datasets allows us to develop a series of novel analyses. They are necessarily illustrative, but by making the data available in a convenient form, we think that readers will be encouraged to make their own analyses of other aspects. In particular, the Gini index serves to highlight unusual features of the biology of a great many transcripts; we refer to this strategy of using the Gini index to analyze expression profiling data as GeneGini.

 © 2018 The Author(s). Published by Elsevier Inc.
This is an open access article under the CC BY license (http://creativecommons.org/licenses/by/4.0/).

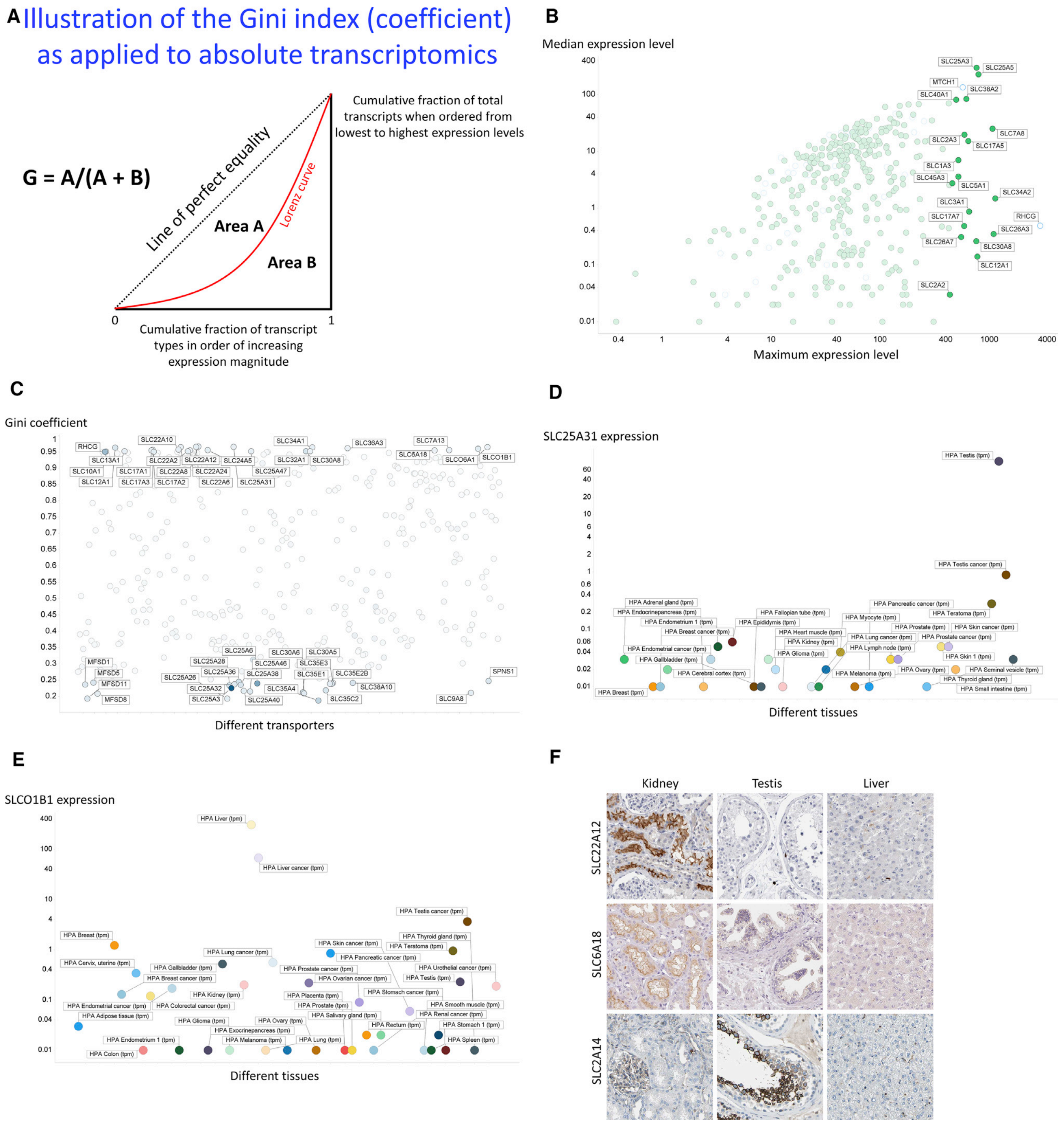

Figure 1. Overall Assessment of Variation in Gene Expression Profiles

(A) The Gini index. Many equivalent definitions are possible. In the usual form, the Gini coefficient is defined mathematically based on the Lorenz curve, which plots the proportion of the total income or wealth of a population (ordinate) that is earned cumulatively by the bottom x% of the population (see diagram) as x increases. Here "income" is the percentage of total transcripts, while the "population" is the individual transporter transcripts considered at one time. (The same general form results if the abscissa is reversed, starting with the top earners, where it takes on the appearance of the more familiar receiver-operator characteristic curve or ROC curve; Baker, 2003; Broadhurst and Kell, 2006; Linden, 2006.) The line at 45° represents uniform expression of each transcript. The Gini coefficient can then be seen as the ratio of the area that lies between the line of equality and the Lorenz curve (labeled A in the figure) to the total area under the line of equality (labeled A and B), i.e., G = A/(A + B).

(B) Median and maximum expression levels (ignoring those with undetectable expression even at the median) in the 59 tissues considered.

(C) Gini coefficient for the expression of all SLCs in 59 tissues; those with Gini coefficients above 0.9 or below 0.25 are shown.

(legend continued on next page)

A preprint has been deposited at bioRxiv (O'Hagan et al., 2017).

RESULTS

Gini Index

Our first interest was to provide a convenient method for summarizing the variation in gene expression profiles in different samples (in this case different tissues and cell lines). A variety of means exist to capture variation; however, none of the more common statistical measures captures the full range well, especially including the many zeroes (undetectable expression levels). One that does is the Gini index (Ceriani and Verme, 2012; Gini, 1909, 1912) or Gini coefficient (GC). This is a non-parametric measure that is widely used in economics to describe distributions of incomes between individuals in a given group or political jurisdiction (e.g., country or region) (Kondo et al., 2012; Pickett and Wilkinson, 2015; Wilkinson and Pickett, 2009). As a summary statistic of the entire Lorenz curve (Lee, 1999) (see Figure 1), it is a statistical measure of the degree of variation represented in a set of values. It ranges between 0 (no variation) and 1 (extreme variation, in which all non-zero values are contained in one individual or example). Clearly it can be used to describe the distribution of anything else, e.g., the structural diversity in chemical libraries (Weidlich and Filippov, 2016) (modulo; O'Hagan and Kell, 2017b). It has very occasionally been used in gene expression profiling studies (Ainali et al., 2012; Jiang et al., 2016; Torre et al., 2017; Tran, 2011). However, in each of these latter cases, including a very recent and nicely done example on cancer cell susceptibility to drugs (Shaffer et al., 2017), where it varied from 0.05 to 1, the Gini index was used for choosing subsets of transcripts that differentiate rare cell types or diseases. Here we *know* the cell types, and the novelty of GeneGini lies in using the Gini index to assess individual genes in terms of the uniqueness of their expression levels. A more intuitive, graphical illustration is given in Figure 1A.

Variation in Expression Profiles of SLCs in Tissues

As is typical in exploratory data analysis (Tukey, 1977), we begin with the following general comments (the full datasets are given in Supplemental Information: Tables S1 and S2):

(1) The variation of transporter expression levels between different tissues or cell lines is very far from being normal (Gaussian) (see Broadhurst and Kell, 2006 for methods; data not shown). The extreme here (and see below) is probably SLCO1B1 (Hagenbuch and Stieger, 2013), whose expression is virtually confined to the liver alone (a fact that has been exploited effectively for drug targeting purposes [Pfefferkorn, 2013]);
(2) The tissue with the maximum overall expression of transporters (SLC and/or ABCs) is the kidney (Σ10,950); that with the fewest is the pancreas (Σ1,490);
(3) The SLCs with, overall, the greatest expression in total are SLC6A15 (a neutral amino acid transporter [Pramod et al., 2013]), whose activity has been implicated in depression (Kohli et al., 2011), and SLC25A3 (a mitochondrial phosphate transporter [Palmieri, 2013]), while that least expressed *in toto* is SLC6A5 (glycine transporter).
(4) Almost every transporter ranges in its expression by over two orders of magnitude in different tissues, and several by more than three or even four orders of magnitude (see also Sreedharan et al., 2011; Winter et al., 2014).
(5) The heatmap of expression levels shows a number of major co-expression clusters.

Figure S1 shows the minimum and maximum expression levels (as TPM [transcripts per million]) for each transporter, with the top 20 (maximum expressions) labeled explicitly. Open circles are those not explicitly labeled as SLC family members. Interestingly, the mitochondrial transporters (Palmieri, 2013) SLC25A3 (for phosphate) and SLC25A5 (for adenine nucleotide translocase [Clémençon et al., 2013]) are among the most highly expressed, as is the non-SLC MTCH1, which, as its name implies, is a mitochondrial carrier homologue. The co-expression of SLC25A3 and SLC25A5 is entirely logical (not shown, but see data files), as ATP synthesis and export require the transport of equimolar amounts of its substrates. Many other SLC25 (mitochondrial transporter) family members are well represented as high expressers in at least one tissue. Note that expression levels below 0.01 TPM are not shown. Figure 1B shows similar data for the median versus the maximum expression in the different tissues, which again serves to highlight the considerable heterogeneity of expression. The median of the set of median expression levels for all the SLCs was 3.19 TPM. In addition, it is not at all the case that a transporter tends to be either highly expressed or weakly expressed; although as many transporters are widely distributed, there is a considerable degree of specialization (see also Sreedharan et al., 2011).

The Gini index for the variation in (inequality of distribution of) transporters (Figure 1C) is fully consistent with this, with a significant number having an exceptionally high value (66 at 0.9 or above), not least SLC22 family members, often in the kidney (see below), and with only 23/409 SLCs having a GC below 0.25. One interpretation is that, mostly, individual transporters may be quite specialized; another is that different tissues require different amounts of specific substrates, although such large differences are thereby not easily explained in general. The *median* GC for this overall class of SLCs and related transporters is 0.587. A number of those with the lowest GCs are again in the SLC25 (mitochondrial transporter) family; this is not unreasonable, since every cell is likely to have mitochondria, but some family members are clearly very specialized for particular mitochondria. Thus (Figure 1D) SLC25A31 (AAC4), a particular isoform of the adenine nucleotide translocase (Palmieri, 2013), is essentially expressed only in the testes (Dolce et al., 2005) (GC = 0.965), a finding of unknown biological significance (Hamazaki et al., 2011). However, since its removal inhibits spermatogenesis (Brower et al., 2007), and thus causes infertility

(D) SLC25A31 is almost exclusively expressed in the testes (the expression levels for others being 100 times less).
(E) SLCO1B1 is almost exclusively expressed in the liver (with the expression level in other tissues being 100 times lower or less).
(F) Antibody-based expression of the SLC22A12, SLC6A18, and SLC2A14 transporters in kidney, testis, and liver tissues. SLC22A12 and SLC6A18 are expressed in renal proximal tubules, whereas SLC2A14 is expressed in cells in seminiferous ducts. Image edge length is 320 μm.

(Brower et al., 2009), it is potentially a target for the development of male contraceptives. Thus, SLCs with very high GCs may provide very tissue-specific targets.

SLCO1B1 (a major transporter of so-called statins) is confined essentially to expression only in the liver (Figure 1E), and its GC is ∼0.96. By contrast (GC = 0.188), transporters such as SLC35A4 are almost universally expressed at a similar level (Figure S2). However, this is not true of all SLC35 family members, since SLC35F2 enjoys a very wide distribution of expression levels in both tissues (Figure S3) and cell lines (Winter et al., 2014). We also have an interest in the ergothioneine transporter (SLC22A4, previously known as OCTN1) (Gründemann et al., 2005), as an example of a transporter that definitely favors the transport of an *exogenous* substrate (O'Hagan and Kell, 2017b); Figure S4 shows its expression profile distribution in the tissues considered; its GC is 0.502. Finally, we illustrate (Figure 1F) the spatial expression of SLC22A12 (URAT1, a urate transporter) (Koepsell, 2013) in the kidney, virtually the only tissue in which it shows expression (Gini index = 0.978). Biologically this implies that uric acid is to be seen more as a product than as a substrate here.

One hypothesis around transporters might be that major nutrient transporters (Palm and Thompson, 2017) might be more universally expressed, since such substrates are nominally available via the bloodstream to most tissues. However, this does not seem to hold up, and the GC again provides a convenient means of clarifying that. Thus, SLC6A18, a neutral amino transporter, has the 15th highest GC (0.955), and its expression is essentially confined to the kidney proximal tubule. Similarly, SLC2A14, a glucose transporter (Mueckler and Thorens, 2013), has a GC of 0.853 and is again largely confined to the testes (Figure 2I). Mueckler and Thorens (2013), however, comment that its physiological substrate is unknown, despite it having 95% sequence identity to the *SLC2A3* gene that definitely encodes a glucose transporter.

Correlations and Heatmaps

Some unexpected correlations arise, e.g., that between the expression of SLC39A5 (ZIP5, a Zn^{2+} transporter [Jeong and Eide, 2013]) and SLC17A4 (supposedly a sodium/phosphate transporter in the vesicular glutamate transport family, of unknown function [Reimer, 2013]; r^2 = 0.86) (Figure 2A). Such findings raise many questions but provide few present answers. However, they do provide useful starting points for the testing of biological hypotheses. In this case, one might hypothesize that they are co-regulated, and indeed both are downregulated during a *Clostridium difficile* infection (Carter et al., 2015).

Co-clustered heatmaps of expression levels provide a convenient visual summary of large amounts of data. Thus, Figure 2B shows the full heatmap for SLC expression in tissues. Although, as stated, all the data are provided in full (Supplemental Information) to allow readers to explore them, we have marked four major clusters (zoomed in in Figures S5–S8). With the exception of a slight preponderance of families SLC 25 and 35 in cluster 3 (Figure S7) and of SLC35 in cluster 4 (Figure S8), there was no obvious clustering at the level of families. This gives weight to the idea that SLC transporters have mainly exhibited *divergent* evolution (Höglund et al., 2011).

SLCs in Cell Lines

Figure 3A shows the minimum non-zero versus maximum expression levels of SLCs in cell lines (Figure 3A). The trends are broadly similar, with some of the most highly expressed transporters again being SLC25A3, SLC25A5, MTCH1, and SLC3A2, although there are also differences. The overall spread seems broadly similar to those of tissues, with a preponderance of transporters having minima in the decade 1–10 TPM and maxima in the decade 20–200 TPM. In this sense, cell lines are a reasonable representation of the behavior of tissues. The number of SLCs with a GC over 0.9 is 70, while those with GCs below 0.25 is 35 (Figure 3B). These numbers and behaviors are also close to those for tissues. The median GC for SLCs in cell lines (0.595) is very close to that for tissues (0.587). We note that there may be a mixture of cell types in the tissues, and that some (or even many) transporters likely exhibit a cell-type-specific expression pattern such as SLC22A12, SLC6A18, and SLC2A14 (Figure 2I). Finally (Figure 3C) we show the extensive (4,000-fold) variation in expression profiles of SLC22A4 (the ergothioneine transporter) in the different cell lines, again illustrating very substantial differences in ‘‘need’’ for this exogenous antioxidant (Halliwell et al., 2016) compound. Consistent with this, the cell line with the greatest expression is a skin cell line, that is normally exposed to atmospheric oxygen.

ABC Transporters in Tissues

Figure 4A shows the minimum and maximum expression levels for all 48 ABCs, many of which lack detectable expression in at least one tissue type. Again, the ranges of expression are considerable, but their expression levels tend to be slightly lower than those of the SLCs. The total numbers are small, but no family (encoded in color in Figure 5A), except possibly F, seems especially highly expressed. The overall most highly expressed ABC transporter is ABCC4. The GCs (Figure 4B) vary more than those of the SLCs, and have a median value of 0.496. Five of 48 GCs are greater than 0.9, while four are below 0.25. Several ABCs exhibit very high GCs, that (0.939) of ABCG5 being the largest; it is mainly expressed in the duodenum and the liver. Those of the F family, however, while highly expressed, also have a low GC, indicating that they tend to be among the more highly expressed in most tissues. Indeed, consistent with their being outliers, they are probably not in fact transporters (e.g., Nishimura et al., 2007).

ABC Transporters in Cell Lines

Figure 4C shows the minimum and maximum expression levels for all 48 ABCs, many of which lack detectable expression in at least one cell line. Again, the ranges of expression are considerable, and somewhat more so than those of the SLCs in tissues. No family (encoded in color in Figure 4C) seems especially highly expressed. The overall most highly expressed ABC transporter is ABCE1. The GCs (Figure 4D) are also larger and vary more than those of both the SLCs and of the ABCs in tissues, with a median value of 0.692, suggesting adaptive selection for specialized purposes in the relevant cell lines. Eleven of 48 GCs are greater than 0.9, while five are below 0.25. Several ABCs exhibit very high GCs, that (0.964) of ABCG5 (a sterol transporter [Kerr et al., 2011]) again being the largest; here it is effectively expressed only in the HepG2 liver carcinoma cell line.

Overall, the median expression levels for SLCs are 3.27 and 1.26 TPM for tissues and cell lines, respectively, while those

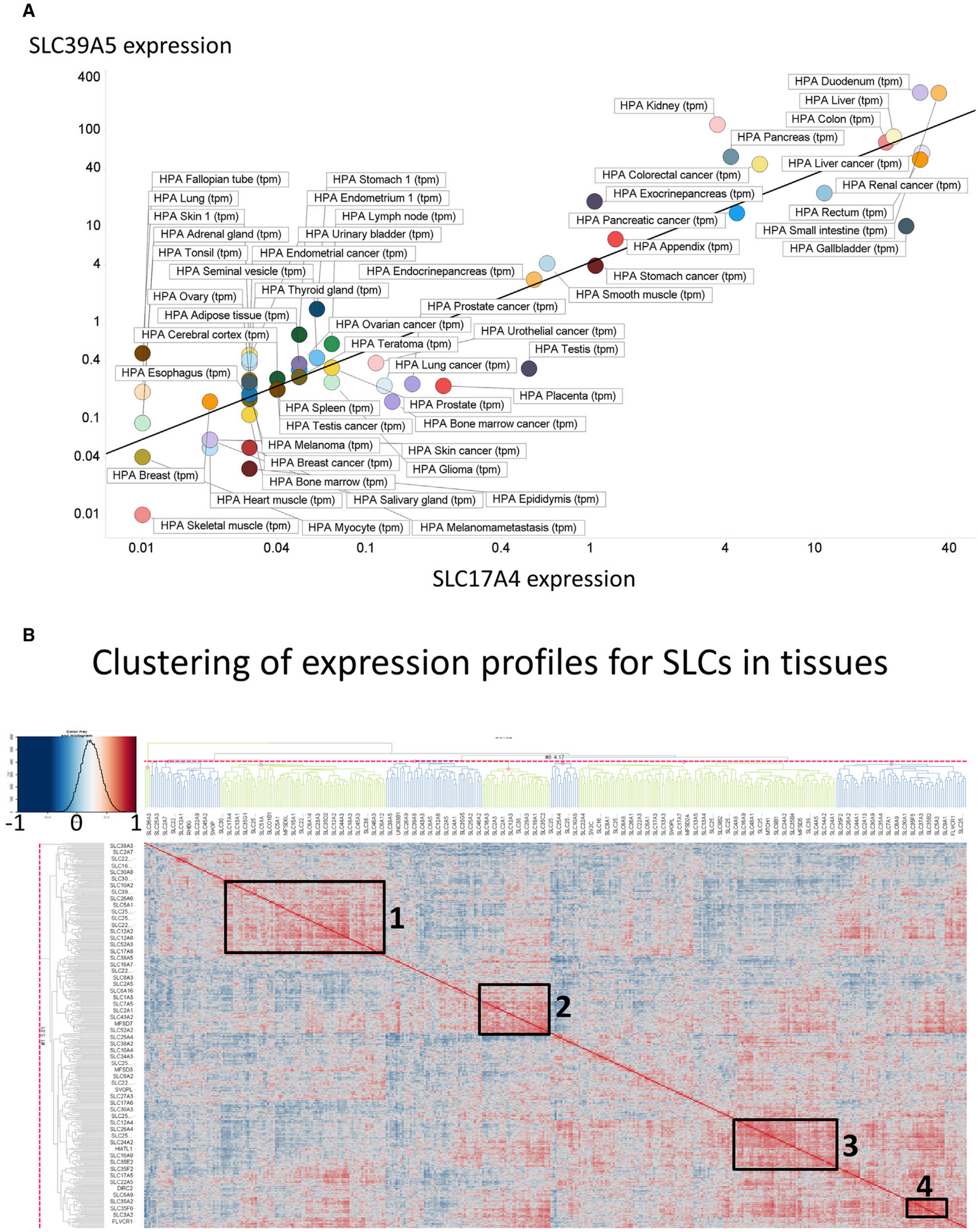

Figure 2. Clustering of (Co-)Expression Profiles of SLC Transporters

(A) Significant correlation (in log-log space) between the expression profiles of SLC39A5 and SLC17A4 (r^2 = 0.86).

(B) Overall heatmap, with four major clusters highlighted.

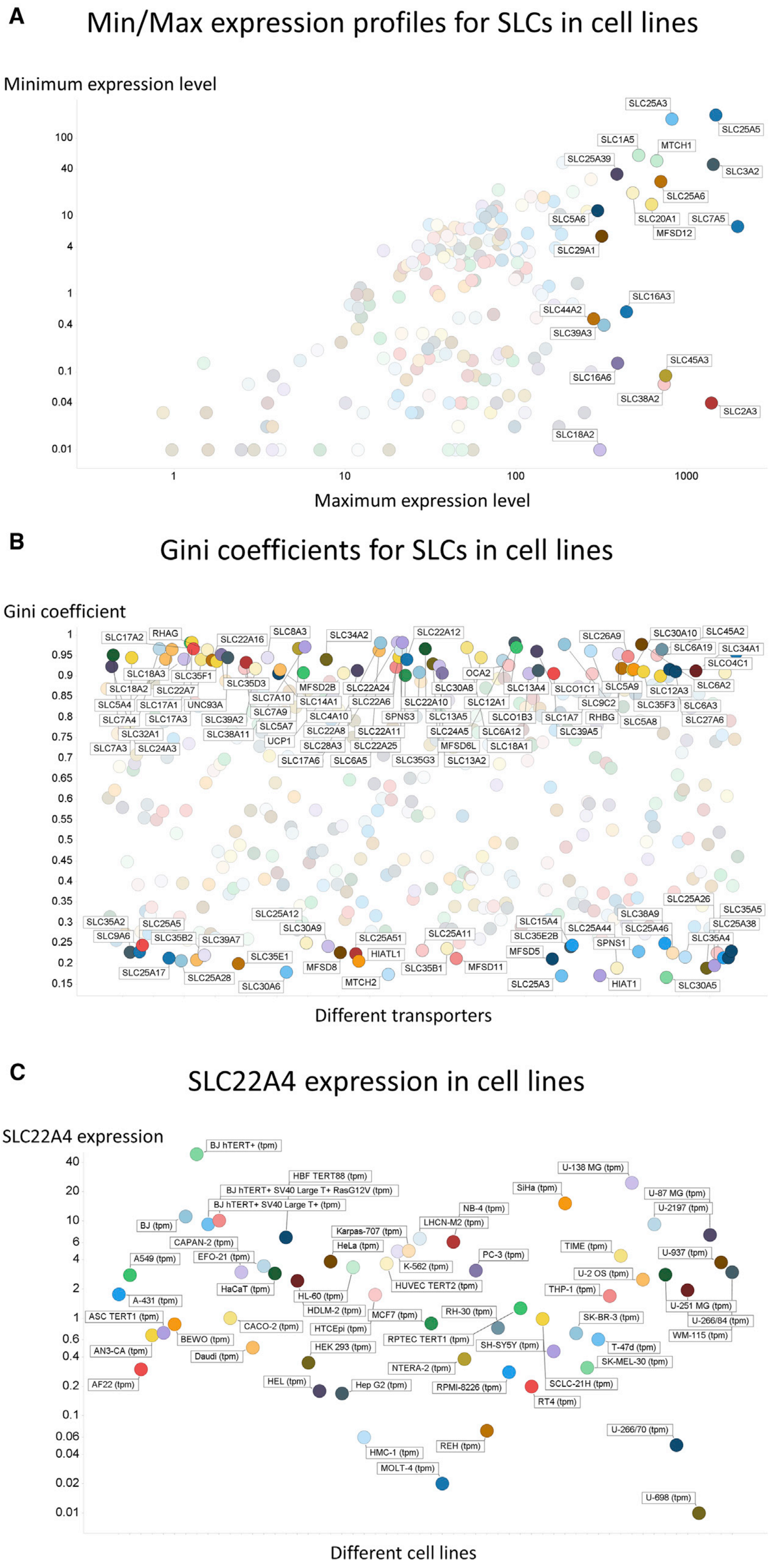

Figure 3. Expression Profiling of Various Transporters in 56 Cell Lines

(A) Minimum and maximum expression levels (as in Figure S1 not showing those with undetectable expression) in the 56 cell lines considered.

(B) Median and maximum expression levels (ignoring those with undetectable expression even at the median) in the 56 cell lines considered.

(C) SLC22A4 expression levels (in TPM) in different cell lines.

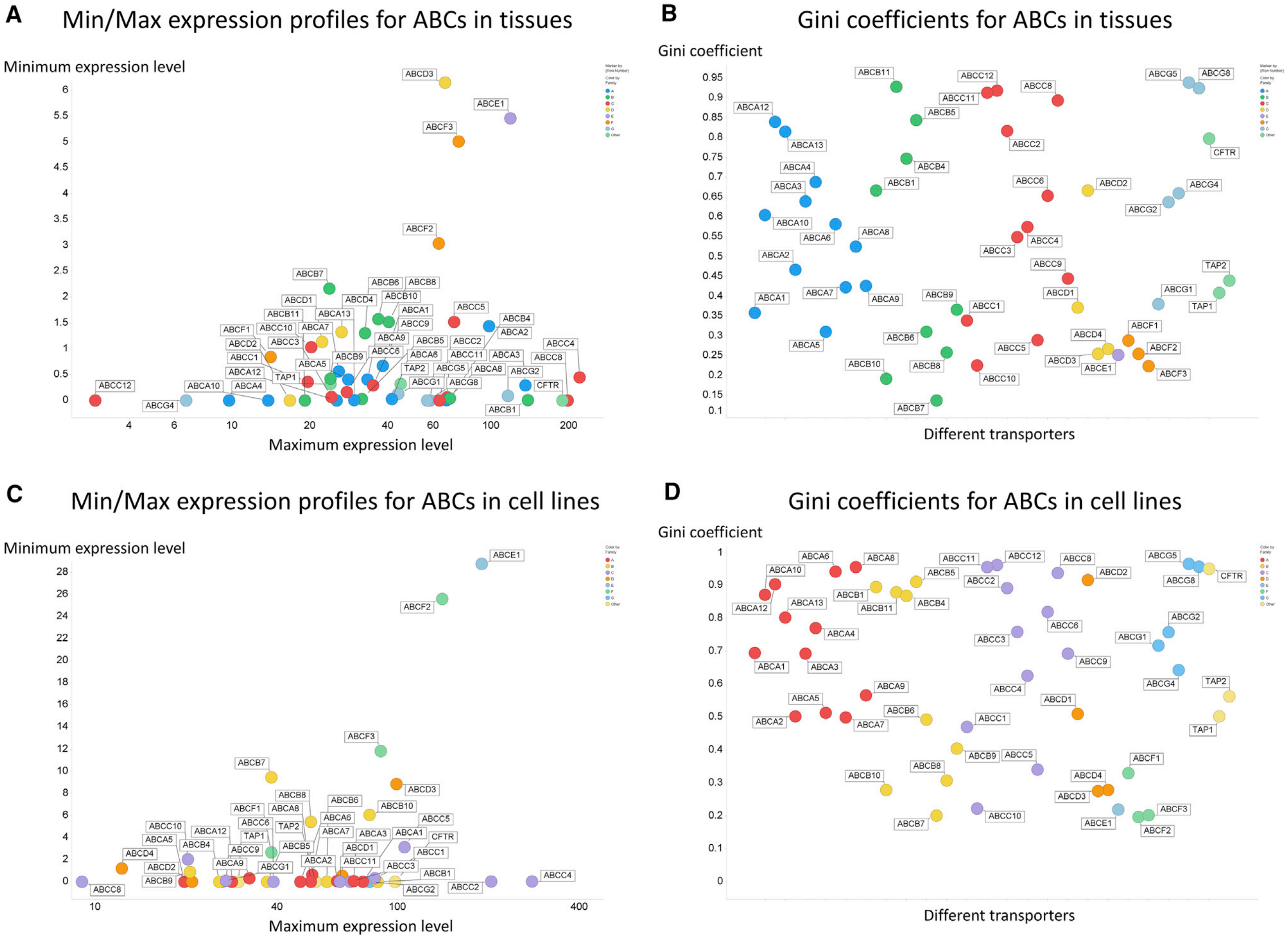

Figure 4. Expression Profiling of Various ABC Transporters in 59 Tissues and 56 Cell Lines
(A) Minimum and maximum expression levels in the 59 tissues considered.
(B) Gini coefficient for the expression of all ABC transporters in 59 tissues.
(C) Minimum and maximum expression levels in the 56 cell lines considered.
(D) Gini coefficient for the expression of all ABC transporters in 56 cell lines.

for ABCs are 4.23 and 1.48 TPM. Thus, while many of these cell lines are cancer derived, the majority of differentially expressed genes (as transporters are) are downregulated in cancer cells (Danielsson et al., 2013). By contrast, if (as helpfully pointed out by a referee) we consider maxima, the median of the maxima in cell lines is close to double that in tissues, both for SLCs (646 versus 368 TPM) and ABCs (98 versus 48 TPM). Thus some transporters are indeed substantially overexpressed in cancer cell lines.

Overall Analysis and Clustering of Cell Lines Based on Transporter Transcripts

Although the data are far from being normally distributed, it is of interest to see which tissues and cell lines are most different from each other based solely on the expression profiles of their transporters; these data (normalized to unit variance) are given as a principal components plot in Figures 5A and 5B, where tissue type is encoded by color, and in the former, whether it is a tumor (gray) or not, is also encoded by a circular shape. Only a small amount of the variance is explained by the first two principal components, consistent with the high variability between tissues and cells, and scree plots are given as insets. The cell line expressing the largest total amount of transporter transcripts (11,566 TPM) *in toto* is BeWo (a placental carcinoma), while that expressing the fewest (5,215 TPM) is ASC TERT1 (a human telomerase-immortalized human adipose-derived mesenchymal stem cell line); the variance in transcripts that may be observed between these two cell lines is given in Figure 5C, with several of those with the greatest differences illustrated. That the total variation in transporter expression is just 2-fold shows (1) the limitation of membrane “real estate” area that partly controls membrane protein expression (Kell et al., 2015), and (2) their overall importance to the cellular economy.

Unusually Heterogeneous Nature of Cell Transporter Expression Profiles

Tissues

While the values of GC for the expression profiles of transporters between different tissues and cells tend to be unusually high, we

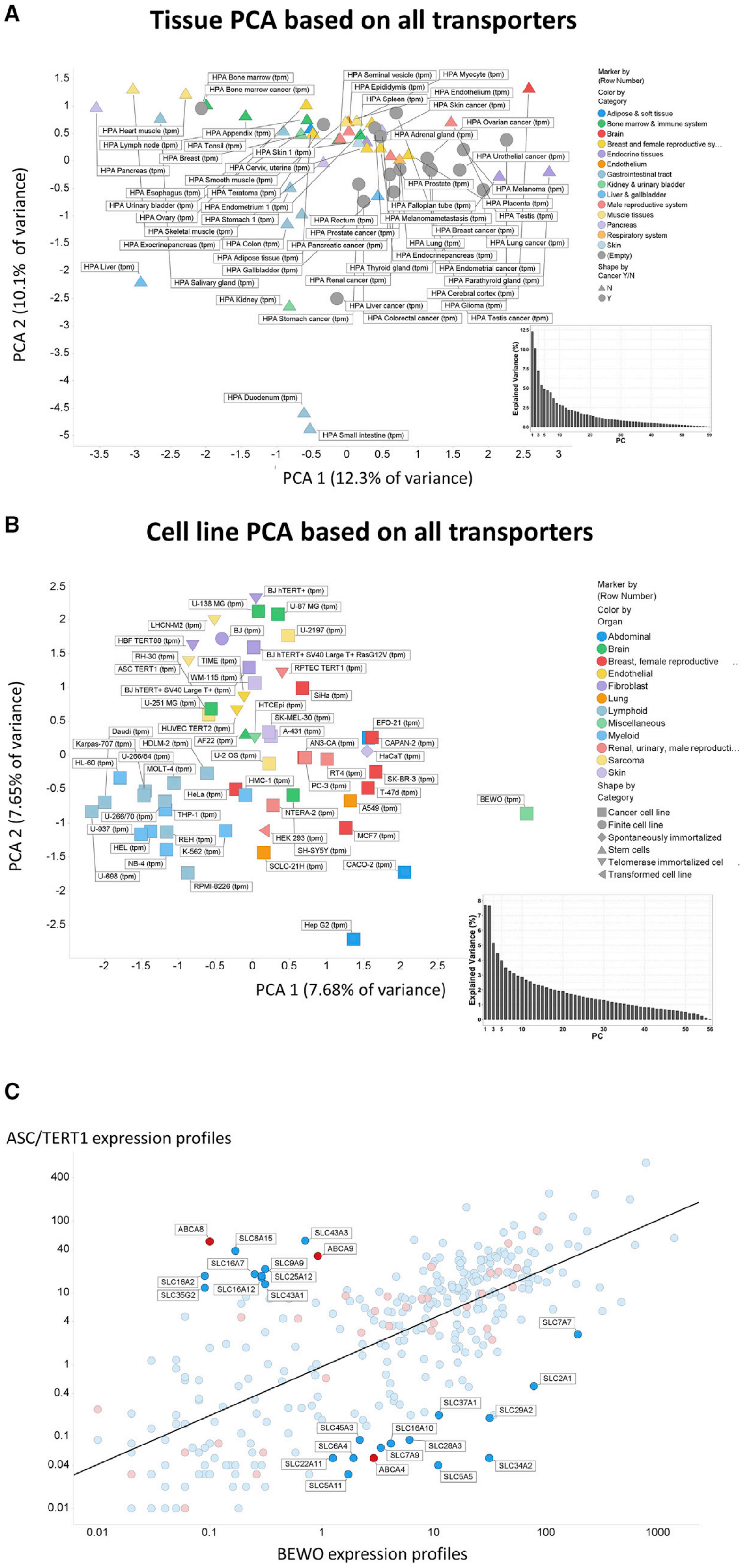

Figure 5. Overall Variance of SLC plus ABC Transporter Expression in Different Tissues, A, and Different Cell Lines, B

(A and B) Analyses were run in KNIME using the expression profiles of both SLCs and ABCs, each normalized to unit variance. Inserts in (A and B) represent the scree plots of percent variance explained by different principal components (PCs).

(C) Variance in transcript levels of both SLC (blue) and ABC (red) transporters in just two cell lines (BEWO and ASC/TERT1) ($r^2 = 0.50$).

have not yet quantified their differences relative to those of other genes.

From such data, the most transcribed gene over any other in cell lines is the *ATP6* gene (mitochondrial ATP synthase subunit a, UniProt P00846, 42,706 TPM in HeLa cells), while that in tissues is *ALB* (albumin, UniProt P02768, 105,947 TPM in liver). The median of all the maxima for tissues is 46 TPM, and for cell lines 40 TPM. Obviously the first of these (*ATP6* and *ALB*) are much larger numbers than those for any transporters (Figures 1 and 4), but the medians (see also Figure 1B) are in quite a similar range; this again illustrates the rather specialist nature of different tissue expression profiles.

The overall picture of the distribution of tissue GCs between the three classes of molecule (SLC/ABC/other) is given in Figure 6 (422 genes had very little expression at all [max = 0.25 TPM] and were ignored). Gene names are in alphabetic order, so it is clear where most of the ABCs (in blue) and SLCs (red) lie. Simply by inspection of this figure we can tell that many more "other" genes (19%) have a GC below say 0.25 than those for SLCs (9%) and ABCs (10%). In a similar vein, 33% of SLCs and 24% of ABCs have a GC exceeding 0.75, while 24% do for other genes. This latter high number is because of several clusters that are visible (and marked) in Figure 6A, specifically those for olfactory receptor proteins (over 300 genes, expressed in specific tissues, which, given their high GCs, necessarily varied for different olfactory receptor proteins) and keratin (over 150 genes, mainly in the melanoma tissues, of which 58 are KRT for keratin and 58 KRTAP for keratin-associated proteins). Note, however, that the maximum expression level for most ORs, and for 69% of the 94 KRTAP (keratin-associated protein) genes, was mainly less than 1 TPM; it is thus uncertain whether they encode detectable levels of protein. By contrast, transcriptional activators in the form of zinc-finger proteins (over 500 transcripts, 82%/97% of which had a median/maximum expression greater than 1 TPM) have very low GCs as they seem to play regulatory roles in almost all cells. Cyclins are of interest, as these should be expressed only in dividing cells. Thus *CCNA1*, the gene for cyclin A1, has a GC of 0.844. However, because our focus here is on transporters, we shall not pursue all these other very interesting questions here.

Genes with Low Expression Profiles as Candidate "Housekeeping" Genes

A variety of genes have previously been proposed as housekeeping or reference genes (Bustin et al., 2009; de Jonge et al., 2007; Gur-Dedeoglu et al., 2009; Hoerndli et al., 2004; Li et al., 2009; Ohl et al., 2005; Oturai et al., 2016; Silver et al., 2006; Tatsumi et al., 2008; Vandesompele et al., 2002; Wang et al., 2010; Zampieri et al., 2010).

However, the expression of most so-called housekeeping genes (that are at least *expressed* in all tissues) actually varies quite widely between tissues (e.g., de Jonge et al., 2007; Eisenberg and Levanon, 2003; Lee et al., 2002; Robinson and Oshlack, 2010); indeed they are sufficiently different that they can be used to classify different tissues (Hsiao et al., 2001)! Here, the housekeeping genes with the lowest GCs, hence those possibly best for normalizing transcriptome or proteome experiment, are FAM32A (an RNA-binding protein; GC = 0.137), ABCB7 (a mitochondrial heme/iron exporter; GC = 0.137), MRPL16 and MRPL21 (mitoribosomal proteins; GC = 0.138), and PCBP1 (an oligo-single-stranded-dC-binding protein; GC = 0.139). Clearly their ubiquitous distribution speaks to their essentiality, and it is certainly of interest that mitoribosomal proteins have such ubiquitous expression, being somewhat equivalent to the 16S rRNA genes widely used in microbial taxonomy and metagenomics. Most of the other 49 large (MRPLxx) and 30 small (MRPSxx) ribosomal protein subunits also had low GCs; others with a GC of 0.15 or below are illustrated in Figure 6B, which also serves to show that most low-Gini gene products have median expression levels in the decade 20–200 TPM (so it is not a strange low-expression artifact).

We note that Eisenberg and Levanon (2013) provide a list of candidate housekeeping genes based on earlier RNA sequencing data. This provides a valuable benchmark for comparison with our approach. However, their list (see http://www.tau.ac.il/~elieis/HKG/HK_genes.txt) consists of no fewer than 3,804 genes (out of the ~25,000 human genes), but provides no quantification of either how good they are as housekeeping/reference genes or of their typical expression levels. Finding the best 6 or 7 out of such an unranked list of 3,804 is a combinatorial problem that would require testing 4.10^{18} or 2.10^{21} combinations, respectively. By contrast we provide *both* the rank order (*and* its justification via the Gini index) *and* the transcription level. Secondly, the paper itself (Eisenberg and Levanon, 2013) used only 16 (not, as here, 59) tissues, and *no* cell lines. Thirdly, the paper does contain a Table of eleven "genes proposed for calibration", representing (on an unstated basis) "a short list of highly uniform and strongly expressed genes that may be used for calibration in future experimental settings"; Table S3 lists these, together with their correct names, UniProt ID, and (from our data) Gini index and median tissue expression levels.

It is rather obvious (Table S3) that the choices in this Table are far poorer than those we suggest in terms of both GC (only one has a GC below 0.15 [for tissues we show 23] Figure 6B) and expression level (e.g., PCBP1 has a GC of 0.139 and an expression level of 209 TPM in tissues).

Indeed, the GCs of other gene products commonly used by experimental biologists to normalize expression profiles were often considerably larger (Table S4), although the more recently proposed *CTBP1* (C-terminal-binding protein 1, UniProt Q13363; 0.204) and GOLGA1 (Golgin subfamily A member 1, UniProt Q92805; 0.189) (Lee et al., 2007) both seem like much better choices. However, the lowest GCs in tissues are FAM32A, ABCB7, MRPL21, and PCBP1 (GC = 0.137–0.139), while the lowest three in cell lines are SF3B2, NXF1, and RBM45 (GC = 0.115–0.122). PCBP1 is both reasonably highly expressed and has a low GC in both tissues (0.139) and cell lines (0.135), and is an excellent novel housekeeping gene. While reference genes are often chosen to be stably expressed across variants of the same cell type rather than across different cells, our very low GC *between* cell types suggests that the GC is indeed a novel and effective way of identifying very useful housekeeping or reference genes in expression profiling studies.

While there was no relationship between the GC and the maximum expression (not shown), there was an interesting inverse relationship between the GC and the median expression level over all genes (Figure 6C), where the correlation coefficient was 0.62. Clearly the exact correlation is also likely to depend on the value of the GC, where at higher levels the Lorenz curve

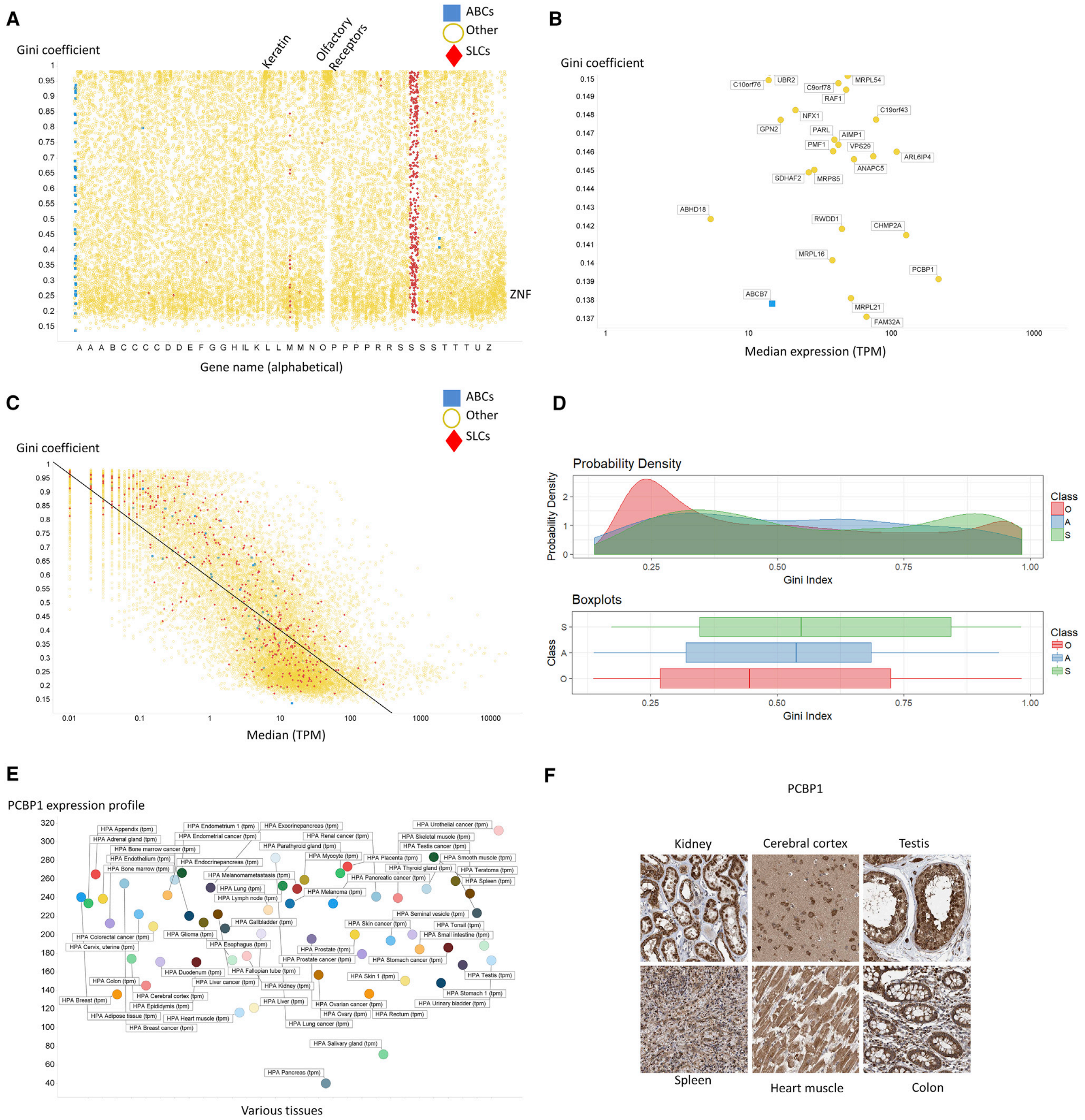

Figure 6. Variation of Gini Coefficients of Different Protein Classes in 59 Tissues

(A) All transcripts, alphabetically, in tissues.

(B) Transcripts with a particularly low Gini coefficient in tissues.

(C) Inverse relationship between Gini index and median expression level in tissues.

(D) Distribution of Gini coefficients in the three classes of transcript in tissues.

(E) Low-Gini PCBP1 expression in tissues.

(F) Antibody-based assessment of the expression of SLC22A12 in a variety of tissues. Image edge length is 320 μm.

(Figure 1) can become highly nonlinear. The overall distribution of GCs for the three classes of protein (SLC/ABC/other) is given in Figure 6D. Finally, because it was one of the gene products with the lowest GC, as well as having a reasonable expression level (median over 100 TPM), in both tissues and cell lines, we show the tissue expression profile of PCBP1 (an intronless gene; Makeyev et al., 1999) in Figure 6E; the overall variation of the great majority of these transcripts is within a 2-fold range. We

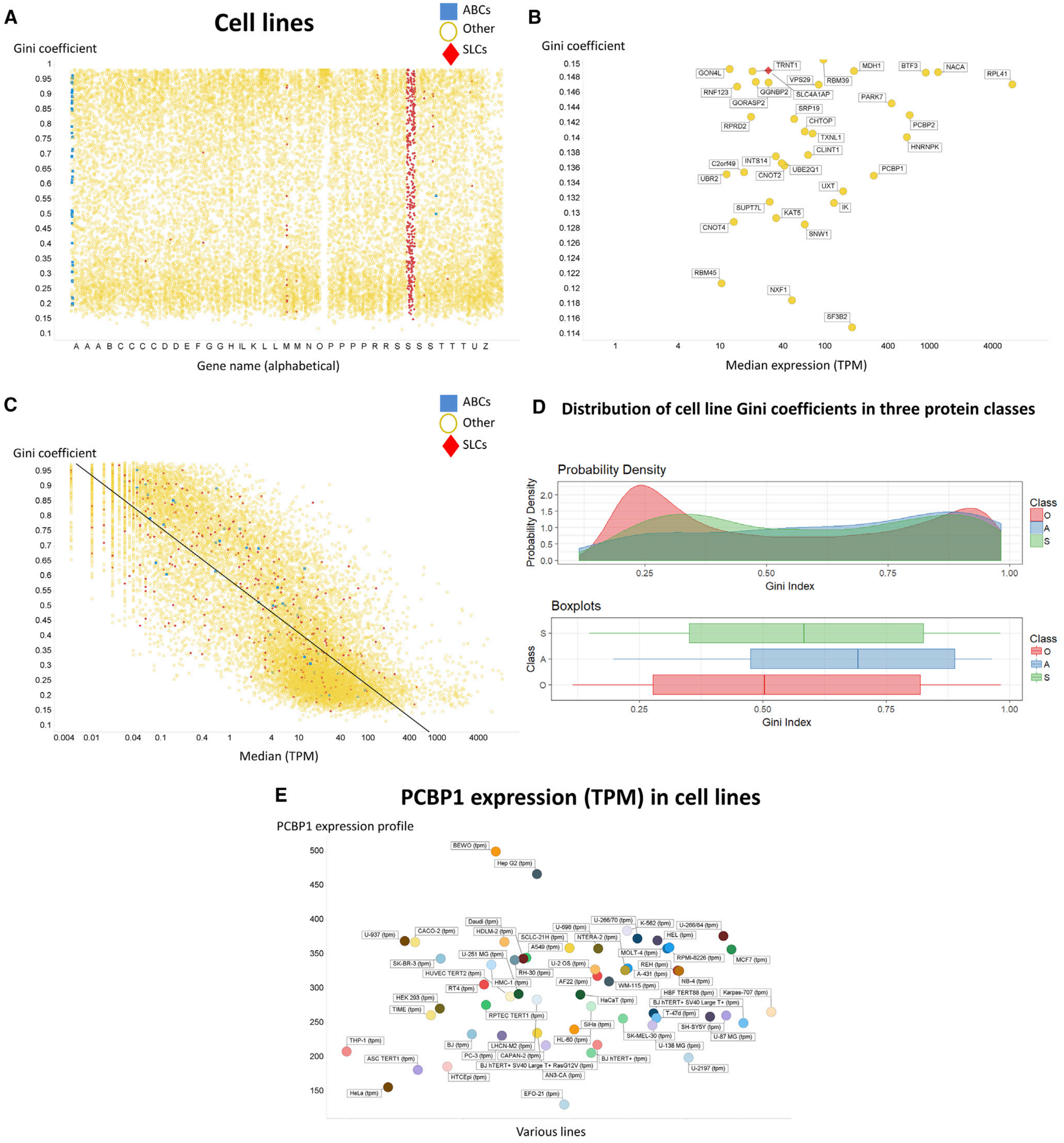

Figure 7. Variation of Gini Coefficients of Different Protein Classes in 56 Cell Lines
(A) All transcripts, alphabetically, in cell lines.
(B) Transcripts with a particularly low Gini coefficient in cell lines.
(C) Inverse relationship between Gini index and median expression level in cell lines.
(D) Distribution in cell lines of Gini coefficients in the three classes.
(E) PCBP1 expression in different cell lines.

also illustrate its distribution in several tissues in Figure 6F. This makes a very strong case for it being a highly useful reference or housekeeping gene.

Cell Lines

The overall data are broadly similar for cell lines (Figure 7A, although the expression of zinc fingers is less homogeneous

than in the tissues). However, the genes with the lowest GC (Figure 7B) are mostly very different from those in tissues. Note that SLC4A1AP that appears is an adaptor protein for SLC4A1 (a chloride-bicarbonate exchanger, commonly known as band 3 protein), so it is not itself a true SLC (and it did not appear in Figure 3B). The gene whose expression showed the very lowest GC, *SF3B2* (UniProt Q13435), is a subunit of an RNA splicing factor, while NXF1 (UniProt Q9UBU9) is a nuclear export factor, and RBM45 (UniProt Q8IUH3) an RNA binding protein 45. It is entirely reasonable that these might be expressed in all cells, and evidently at a fairly constant level. Overall, we conclude that the GeneGini approach is capable of finding novel housekeeping genes to act as references for microarrays and for qPCR, and will be particularly beneficial in studies employing several differentiated cell/tissue types. There is again a correlation between the Gini index and median expression level (r^2 = 0.67) (Figure 7C). Overall, we find that 8.5% of SLCs, 16% of ABCs (including two F-family members), and 18% of other genes have a GC below 0.25, while those above 0.75 are ABC 32%, SLC 25%, and other 19%. Again, there is a significantly greater heterogeneity among transporter genes than among other genes when taken as a whole (Figure 7D). Finally, Figure 7E shows the expression profile of PCBP1 in cell lines; again the overwhelming majority is within a 2-fold range, indicating its excellent candidature as a novel reference gene.

Discussion

The present paper has highlighted at least three main areas. First, we exploit the GC as a novel, convenient, and easily understandable metric for reflecting how unequally a given transcript is expressed in a large series of tissues or cell lines. In contrast to its usual use in economics, where it ranges from ~0.25 to ~0.51 in different countries, the Gini index here ranged from as low as 0.11 to as high as 0.98, reflecting in the latter case virtually unique expression in a particular tissue. In many cases, the biology underpinning this is quite opaque, but the purpose of data-driven studies is to generate rather than to test hypotheses (Kell and Oliver, 2004). We also recognize here that we have paid relatively little attention to the distribution of transporters *within* different tissues and their potential cell-type-specific distribution within an organ (e.g., Bahar Halpern et al., 2017), where they presumably account for the very striking intra-organ distributions of drugs (e.g., Römpp et al., 2011); that will have to be a subject for further work.

A second chief area of interest is the distribution of transporters between different tissues. A detailed analysis showed that they tended to have significantly higher GCs than did other gene families. This illustrates the point that despite the fact that their substrates are almost uniformly available via the bloodstream, and biochemistry textbooks and wallcharts largely show this, they clearly use substrates differentially (ergothioneine and the SLC22A4 transporter being a nice example; Gründemann et al., 2005). It also implies strongly that in many cases we do not in fact know the natural substrates, many of which are clearly exogenous (O'Hagan and Kell, 2017b).

The third main recognition is that the Gini index provides a particularly useful, convenient, non-parametric, and intelligible means of identifying those genes whose expression profile varies least across a series of cells or tissues, thus providing a novel and convenient strategy for the identification of those reference or housekeeping genes best used as genes against which to normalize other expression profiles in a variety of studies. We have here highlighted quite a number that have not previously been so identified.

Overall, we consider that assessing the Gini index for the distribution of particular transporters and other proteins between different cells has much to offer the development of novel biology; it should prove a highly useful addition to the armory of both the systems biologist and the data analyst.

STAR★METHODS

Detailed methods are provided in the online version of this paper and include the following:

- KEY RESOURCES TABLE
- CONTACT FOR REAGENT AND RESOURCE SHARING
- METHOD DETAILS
  - Gini Index
  - Minimum and Maximum Expression Profiles
- QUANTIFICATION AND STATISTICAL ANALYSIS
- DATA AND SOFTWARE AVAILABILITY

SUPPLEMENTAL INFORMATION

Supplemental Information includes eight figures and four tables and can be found with this article online at https://doi.org/10.1016/j.cels.2018.01.003.

ACKNOWLEDGMENTS

D.B.K. and P.J.D. thank the BBSRC (grant BB/P009042/1) for financial support. E.L. thanks the entire staff of the Human Protein Atlas program. E.L. acknowledges financial support by the Knut and Alice Wallenberg Foundation and the Erling Persson Foundation and facility support by the Science for Life Laboratory (SciLifeLab).

AUTHOR CONTRIBUTIONS

The project was initiated following an initial discussion between E.L. and D.B.K. at a scientific conference. D.B.K. highlighted the utility of the GC, and produced many of the visualisations. S.O. adapted the Gini method and performed most of the analyses that were done using KNIME. M.W.M. did the principal-component analyses, while P.J.D. contributed in particular to the analysis of the housekeeping genes. E.L. contributed the original data and images, and multiple insights thereto. All authors contributed to the writing and approval of the manuscript.

DECLARATION OF INTERESTS

The authors declare no competing interests.

Received: June 26, 2017
Revised: September 26, 2017
Accepted: December 30, 2017
Published: February 7, 2018

REFERENCES

Ainali, C., Valeyev, N., Perera, G., Williams, A., Gudjonsson, J.E., Ouzounis, C.A., Nestle, F.O., and Tsoka, S. (2012). Transcriptome classification reveals molecular subtypes in psoriasis. BMC Genomics *13*, 472.

Almén, M.S., Nordström, K.J., Fredriksson, R., and Schiöth, H.B. (2009). Mapping the human membrane proteome: a majority of the human membrane

proteins can be classified according to function and evolutionary origin. BMC Biol. *7*, 50.

Halpern, K.B., Shenhav, R., Matcovitch-Natan, O., Toth, B., Lemze, D., Golan, M., Massasa, E.E., Baydatch, S., Landen, S., Moor, A.E., et al. (2017). Single-cell spatial reconstruction reveals global division of labour in the mammalian liver. Nature *542*, 352–356.

Baker, S.G. (2003). The central role of receiver operating characteristic (ROC) curves in evaluating tests for the early detection of cancer. J. Natl. Cancer Inst. *95*, 511–515.

Berthold, M.R., Cebron, N., Dill, F., Gabriel, T.R., Kötter, T., Meinl, T., Ohl, P., Sieb, C., Thiel, K., and Wiswedel, B. (2008). KNIME: the konstanz information miner. In Data Analysis, Machine Learning and Applications, C. Preisach, H. Burkhardt, L. Schmidt-Thieme, and R. Decker, eds. (Springer), pp. 319–326.

Bray, N.L., Pimentel, H., Melsted, P., and Pachter, L. (2016). Near-optimal probabilistic RNA-seq quantification. Nat. Biotechnol. *34*, 525–527.

Broadhurst, D., and Kell, D.B. (2006). Statistical strategies for avoiding false discoveries in metabolomics and related experiments. Metabolomics *2*, 171–196.

Brower, J.V., Lim, C.H., Jorgensen, M., Oh, S.P., and Terada, N. (2009). Adenine nucleotide translocase 4 deficiency leads to early meiotic arrest of murine male germ cells. Reproduction *138*, 463–470.

Brower, J.V., Rodic, N., Seki, T., Jorgensen, M., Fliess, N., Yachnis, A.T., McCarrey, J.R., Oh, S.P., and Terada, N. (2007). Evolutionarily conserved mammalian adenine nucleotide translocase 4 is essential for spermatogenesis. J. Biol. Chem. *282*, 29658–29666.

Bustin, S.A., Benes, V., Garson, J.A., Hellemans, J., Huggett, J., Kubista, M., Mueller, R., Nolan, T., Pfaffl, M.W., Shipley, G.L., et al. (2009). The MIQE guidelines: minimum information for publication of quantitative real-time PCR experiments. Clin. Chem. *55*, 611–622.

Carter, G.P., Chakravorty, A., Pham Nguyen, T.A., Mileto, S., Schreiber, F., Li, L., Howarth, P., Clare, S., Cunningham, B., Sambol, S.P., et al. (2015). Defining the roles of tcda and tcdb in localized gastrointestinal disease, systemic organ damage, and the host response during clostridium difficile infections. MBio *6*, e00551.

Ceriani, L., and Verme, P. (2012). The origins of the Gini index: extracts from *Variabilità e Mutabilità* (1912) by Corrado Gini. J. Econ. Inequal. *10*, 421–443.

César-Razquin, A., Snijder, B., Frappier-Brinton, T., Isserlin, R., Gyimesi, G., Bai, X., Reithmeier, R.A., Hepworth, D., Hediger, M.A., Edwards, A.M., et al. (2015). A call for systematic research on solute carriers. Cell *162*, 478–487.

Chen, Z., Shi, T., Zhang, L., Zhu, P., Deng, M., Huang, C., Hu, T., Jiang, L., and Li, J. (2016). Mammalian drug efflux transporters of the ATP binding cassette (ABC) family in multidrug resistance: a review of the past decade. Cancer Lett. *370*, 153–164.

Clémençon, B., Babot, M., and Trezeguet, V. (2013). The mitochondrial ADP/ATP carrier (SLC25 family): pathological implications of its dysfunction. Mol. Aspects Med. *34*, 485–493.

Colas, C., Ung, P.M.U., and Schlessinger, A. (2016). SLC transporters: structure, function, and drug discovery. Medchemcomm *7*, 1069–1081.

da Cunha, J.P., Galante, P.A., de Souza, J.E., de Souza, R.F., Carvalho, P.M., Ohara, D.T., Moura, R.P., Oba-Shinja, S.M., Marie, S.K., Silva, W.A., Jr., et al. (2009). Bioinformatics construction of the human cell surfaceome. Proc. Natl. Acad. Sci. USA *106*, 16752–16757.

Danielsson, F., Skogs, M., Huss, M., Rexhepaj, E., O'Hurley, G., Klevebring, D., Pontén, F., Gad, A.K.B., Uhlén, M., and Lundberg, E. (2013). Majority of differentially expressed genes are down-regulated during malignant transformation in a four-stage model. Proc. Natl. Acad. Sci. USA *110*, 6853–6858.

de Jonge, H.J., Fehrmann, R.S., de Bont, E.S., Hofstra, R.M., Gerbens, F., Kamps, W.A., de Vries, E.G., van der Zee, A.G., te Meerman, G.J., and ter Elst, A. (2007). Evidence based selection of housekeeping genes. PLoS One *2*, e898.

Dobson, P.D., and Kell, D.B. (2008). Carrier-mediated cellular uptake of pharmaceutical drugs: an exception or the rule? Nat. Rev. Drug Discov. *7*, 205–220.

Dolce, V., Scarcia, P., Iacopetta, D., and Palmieri, F. (2005). A fourth ADP/ATP carrier isoform in man: identification, bacterial expression, functional characterization and tissue distribution. FEBS Lett. *579*, 633–637.

Eadie, L.N., Hughes, T.P., and White, D.L. (2014). Interaction of the efflux transporters ABCB1 and ABCG2 with imatinib, nilotinib, and dasatinib. Clin. Pharmacol. Ther. *95*, 294–306.

Eisenberg, E., and Levanon, E.Y. (2003). Human housekeeping genes are compact. Trends Genet. *19*, 362–365.

Eisenberg, E., and Levanon, E.Y. (2013). Human housekeeping genes, revisited. Trends Genet. *29*, 569–574.

Fredriksson, R., Nordström, K.J., Stephansson, O., Hägglund, M.G., and Schiöth, H.B. (2008). The solute carrier (SLC) complement of the human genome: phylogenetic classification reveals four major families. FEBS Lett. *582*, 3811–3816.

Giacomini, K.M., and Huang, S.M. (2013). Transporters in drug development and clinical pharmacology. Clin. Pharmacol. Ther. *94*, 3–9.

Giacomini, K.M., Huang, S.M., Tweedie, D.J., Benet, L.Z., Brouwer, K.L., Chu, X., Dahlin, A., Evers, R., Fischer, V., Hillgren, K.M., et al. (2010). Membrane transporters in drug development. Nat. Rev. Drug Discov. *9*, 215–236.

Gini, C. (1909). Concentration and dependency ratios (in Italian). Rivista di Politica Economica *87*, 769–789.

Gini, C. (1912). Variabilità e Mutabilità. Contributo allo Studio delle Distribuzioni e delle Relazioni Statistiche (C. Cuppini).

Gründemann, D., Harlfinger, S., Golz, S., Geerts, A., Lazar, A., Berkels, R., Jung, N., Rubbert, A., and Schömig, E. (2005). Discovery of the ergothioneine transporter. Proc. Natl. Acad. Sci. USA *102*, 5256–5261.

Gur-Dedeoglu, B., Konu, O., Bozkurt, B., Ergul, G., Seckin, S., and Yulug, I.G. (2009). Identification of endogenous reference genes for qRT-PCR analysis in normal matched breast tumor tissues. Oncol. Res. *17*, 353–365.

Hagenbuch, B., and Stieger, B. (2013). The SLCO (former SLC21) superfamily of transporters. Mol. Aspects Med. *34*, 396–412.

Halliwell, B., Cheah, I.K., and Drum, C.L. (2016). Ergothioneine, an adaptive antioxidant for the protection of injured tissues? A hypothesis. Biochem. Biophys. Res. Commun. *470*, 245–250.

Hamazaki, T., Leung, W.Y., Cain, B.D., Ostrov, D.A., Thorsness, P.E., and Terada, N. (2011). Functional expression of human adenine nucleotide translocase 4 in *Saccharomyces cerevisiae*. PLoS One *6*, e19250.

Hediger, M.A., Clémençon, B., Burrier, R.E., and Bruford, E.A. (2013). The ABCs of membrane transporters in health and disease (SLC series): introduction. Mol. Aspects Med. *34*, 95–107.

Hoerndli, F.J., Toigo, M., Schild, A., Götz, J., and Day, P.J. (2004). Reference genes identified in SH-SY5Y cells using custom-made gene arrays with validation by quantitative polymerase chain reaction. Anal Biochem. *335*, 30–41.

Höglund, P.J., Nordström, K.J.V., Schiöth, H.B., and Fredriksson, R. (2011). The solute carrier families have a remarkably long evolutionary history with the majority of the human families present before divergence of Bilaterian species. Mol. Biol. Evol. *28*, 1531–1541.

Hsiao, L.L., Dangond, F., Yoshida, T., Hong, R., Jensen, R.V., Misra, J., Dillon, W., Lee, K.F., Clark, K.E., Haverty, P., et al. (2001). A compendium of gene expression in normal human tissues. Physiol. Genomics *7*, 97–104.

Jeong, J., and Eide, D.J. (2013). The SLC39 family of zinc transporters. Mol. Aspects Med. *34*, 612–619.

Jiang, L., Chen, H., Pinello, L., and Yuan, G.C. (2016). GiniClust: detecting rare cell types from single-cell gene expression data with Gini index. Genome Biol. *17*, 144.

Kell, D.B. (2015). The transporter-mediated cellular uptake of pharmaceutical drugs is based on their metabolite-likeness and not on their bulk biophysical properties: towards a systems pharmacology. Perspect. Sci. *6*, 66–83.

Kell, D.B. (2016). How drugs pass through biological cell membranes – a paradigm shift in our understanding? Beilstein Mag. *2*, https://doi.org/10.3762/bmag.5. http://www.beilstein-institut.de/download/628/609_kell.pdf.

Kell, D.B., Dobson, P.D., Bilsland, E., and Oliver, S.G. (2013). The promiscuous binding of pharmaceutical drugs and their transporter-mediated uptake into

cells: what we (need to) know and how we can do so. Drug Disc Today *18*, 218–239.

Kell, D.B., Dobson, P.D., and Oliver, S.G. (2011). Pharmaceutical drug transport: the issues and the implications that it is essentially carrier-mediated only. Drug Discov. Today *16*, 704–714.

Kell, D.B., and Oliver, S.G. (2004). Here is the evidence, now what is the hypothesis? The complementary roles of inductive and hypothesis-driven science in the post-genomic era. Bioessays *26*, 99–105.

Kell, D.B., and Oliver, S.G. (2014). How drugs get into cells: tested and testable predictions to help discriminate between transporter-mediated uptake and lipoidal bilayer diffusion. Front Pharmacol. *5*, 231.

Kell, D.B., Swainston, N., Pir, P., and Oliver, S.G. (2015). Membrane transporter engineering in industrial biotechnology and whole-cell biocatalysis. Trends Biotechnol. *33*, 237–246.

Kerr, I.D., Haider, A.J., and Gelissen, I.C. (2011). The ABCG family of membrane-associated transporters: you don't have to be big to be mighty. Br. J. Pharmacol. *164*, 1767–1779.

Koepsell, H. (2013). The SLC22 family with transporters of organic cations, anions and zwitterions. Mol. Aspects Med. *34*, 413–435.

Kohli, M.A., Lucae, S., Saemann, P.G., Schmidt, M.V., Demirkan, A., Hek, K., Czamara, D., Alexander, M., Salyakina, D., Ripke, S., et al. (2011). The neuronal transporter gene SLC6A15 confers risk to major depression. Neuron *70*, 252–265.

Kondo, N., van Dam, R.M., Sembajwe, G., Subramanian, S.V., Kawachi, I., and Yamagata, Z. (2012). Income inequality and health: the role of population size, inequality threshold, period effects and lag effects. J. Epidemiol. Community Health *66*, e11.

Lee, P.D., Sladek, R., Greenwood, C.M., and Hudson, T.J. (2002). Control genes and variability: absence of ubiquitous reference transcripts in diverse mammalian expression studies. Genome Res. *12*, 292–297.

Lee, S., Jo, M., Lee, J., Koh, S.S., and Kim, S. (2007). Identification of novel universal housekeeping genes by statistical analysis of microarray data. J. Biochem. Mol. Biol. *40*, 226–231.

Lee, W.C. (1999). Probabilistic analysis of global performances of diagnostic tests: interpreting the Lorenz curve-based summary measures. Stat. Med. *18*, 455–471.

Li, Y.L., Ye, F., Hu, Y., Lu, W.G., and Xie, X. (2009). Identification of suitable reference genes for gene expression studies of human serous ovarian cancer by real-time polymerase chain reaction. Anal Biochem. *394*, 110–116.

Lin, L., Yee, S.W., Kim, R.B., and Giacomini, K.M. (2015). SLC transporters as therapeutic targets: emerging opportunities. Nat. Rev. Drug Discov. *14*, 543–560.

Linden, A. (2006). Measuring diagnostic and predictive accuracy in disease management: an introduction to receiver operating characteristic (ROC) analysis. J. Eval. Clin. Pract. *12*, 132–139.

Makeyev, A.V., Chkheidze, A.N., and Liebhaber, S.A. (1999). A set of highly conserved RNA-binding proteins, alphaCP-1 and alphaCP-2, implicated in mRNA stabilization, are coexpressed from an intronless gene and its intron-containing paralog. J. Biol. Chem. *274*, 24849–24857.

Montanari, F., and Ecker, G.F. (2015). Prediction of drug-ABC transporter interaction - recent advances and future challenges. Adv. Drug Deliv. Rev. *86*, 17–26.

Mueckler, M., and Thorens, B. (2013). The SLC2 (GLUT) family of membrane transporters. Mol. Aspects Med. *34*, 121–138.

Nishimura, S., Tsuda, H., Ito, K., Jobo, T., Yaegashi, N., Inoue, T., Sudo, T., Berkowitz, R.S., and Mok, S.C. (2007). Differential expression of ABCF2 protein among different histologic types of epithelial ovarian cancer and in clear cell adenocarcinomas of different organs. Hum. Pathol. *38*, 134–139.

O'Hagan, S., and Kell, D.B. (2015). The KNIME workflow environment and its applications in Genetic Programming and machine learning. Genet. Progr. Evol. Mach. *16*, 387–391.

O'Hagan, S., and Kell, D.B. (2017a). Analysis of drug-endogenous human metabolite similarities in terms of their maximum common substructures. J. Cheminform *9*, 18.

O'Hagan, S., and Kell, D.B. (2017b). Consensus rank orderings of molecular fingerprints illustrate the 'most genuine' similarities between marketed drugs and small endogenous human metabolites, but highlight exogenous natural products as the most important 'natural' drug transporter substrates. ADMET & DMPK *5*, 85–125.

O'Hagan, S., Wright Muelas, M., Day, P.J., Lundberg, E., and Kell, D.B. (2017). Novel 'housekeeping' genes and an unusually heterogeneous distribution of transporter expression profiles in human tissues and cell lines, assessed using the Gini coefficient. bioRxiv, 155697.

O'Hagan, S., and Kell, D.B. (2018). Analysing and navigating natural products space for generating small, diverse, but representative chemical libraries. Biotechnol. J *13*, 1700503.

O'Hagan, S., Swainston, N., Handl, J., and Kell, D.B. (2015). A 'rule of 0.5' for the metabolite-likeness of approved pharmaceutical drugs. Metabolomics *11*, 323–339.

Ohl, F., Jung, M., Xu, C., Stephan, C., Rabien, A., Burkhardt, M., Nitsche, A., Kristiansen, G., Loening, S.A., Radonić, A., et al. (2005). Gene expression studies in prostate cancer tissue: which reference gene should be selected for normalization? J. Mol. Med. (Berl) *83*, 1014–1024.

Oturai, D.B., Søndergaard, H.B., Börnsen, L., Sellebjerg, F., and Christensen, J.R. (2016). Identification of suitable reference genes for peripheral blood mononuclear cell subset studies in multiple sclerosis. Scand. J. Immunol. *83*, 72–80.

Palm, W., and Thompson, C.B. (2017). Nutrient acquisition strategies of mammalian cells. Nature *546*, 234–242.

Palmieri, F. (2013). The mitochondrial transporter family SLC25: identification, properties and physiopathology. Mol. Aspects Med. *34*, 465–484.

Perland, E., and Fredriksson, R. (2017). Classification systems of secondary active transporters. Trends Pharmacol. Sci. *38*, 305–315.

Pfefferkorn, J.A. (2013). Strategies for the design of hepatoselective glucokinase activators to treat type 2 diabetes. Expert Opin. Drug Discov. *8*, 319–330.

Pickett, K.E., and Wilkinson, R.G. (2015). Income inequality and health: a causal review. Social Sci. Med. *128*, 316–326.

Pramod, A.B., Foster, J., Carvelli, L., and Henry, L.K. (2013). SLC6 transporters: structure, function, regulation, disease association and therapeutics. Mol. Aspects Med. *34*, 197–219.

Rees, D.C., Johnson, E., and Lewinson, O. (2009). ABC transporters: the power to change. Nat. Rev. Mol. Cell Biol. *10*, 218–227.

Reimer, R.J. (2013). SLC17: a functionally diverse family of organic anion transporters. Mol. Aspects Med. *34*, 350–359.

Robinson, M.D., and Oshlack, A. (2010). A scaling normalization method for differential expression analysis of RNA-seq data. Genome Biol. *11*, R25.

Römpp, A., Guenther, S., Takats, Z., and Spengler, B. (2011). Mass spectrometry imaging with high resolution in mass and space (HR^2 MSI) for reliable investigation of drug compound distributions on the cellular level. Anal Bioanal. Chem. *401*, 65–73.

Schlessinger, A., Matsson, P., Shima, J.E., Pieper, U., Yee, S.W., Kelly, L., Apeltsin, L., Stroud, R.M., Ferrin, T.E., Giacomini, K.M., et al. (2010). Comparison of human solute carriers. Protein Sci. *19*, 412–428.

Shaffer, S.M., Dunagin, M.C., Torborg, S.R., Torre, E.A., Emert, B., Krepler, C., Beqiri, M., Sproesser, K., Brafford, P.A., Xiao, M., et al. (2017). Rare cell variability and drug-induced reprogramming as a mode of cancer drug resistance. Nature *546*, 431–435.

Silver, N., Best, S., Jiang, J., and Thein, S.L. (2006). Selection of housekeeping genes for gene expression studies in human reticulocytes using real-time PCR. BMC Mol. Biol. *7*, 33.

Sreedharan, S., Stephansson, O., Schiöth, H.B., and Fredriksson, R. (2011). Long evolutionary conservation and considerable tissue specificity of several atypical solute carrier transporters. Gene *478*, 11–18.

Stanley, L.A., Horsburgh, B.C., Ross, J., Scheer, N., and Wolf, C.R. (2009). Drug transporters: gatekeepers controlling access of xenobiotics to the cellular interior. Drug Metab. Rev. *41*, 27–65.

Tatsumi, K., Ohashi, K., Taminishi, S., Okano, T., Yoshioka, A., and Shima, M. (2008). Reference gene selection for real-time RT-PCR in regenerating mouse livers. Biochem. Biophys. Res. Commun. *374*, 106–110.

Thul, P.J., Åkesson, L., Wiking, M., Mahdessian, D., Geladaki, A., Ait Blal, H., Alm, T., Asplund, A., Björk, L., Breckels, L.M., et al. (2017). A subcellular map of the human proteome. Science *356*, https://doi.org/10.1126/science.aal3321.

Torre, E., Dueck, H., Shaffer, S., Gospocic, J., Gupte, R., Bonasio, R., Kim, J., Murray, J., and Raj, A. (2017). A comparison between single cell RNA sequencing and single molecule RNA FISH for rare cell analysis. bioRxiv. https://doi.org/10.1101/138289.

Tran, Q.N. (2011). Improving the accuracy of gene expression profile classification with Lorenz curves and Gini ratios. Softw. Tools Algorithms Biol. Syst. *696*, 83–90.

Tukey, J.W. (1977). Exploratory Data Analysis (Addison-Wesley).

Uhlén, M., Fagerberg, L., Hallstrom, B.M., Lindskog, C., Oksvold, P., Mardinoglu, A., Sivertsson, Å., Kampf, C., Sjöstedt, E., Asplund, A., et al. (2015). Tissue-based map of the human proteome. Science *347*, 1260419.

Vandesompele, J., De Preter, K., Pattyn, F., Poppe, B., Van Roy, N., De Paepe, A., and Speleman, F. (2002). Accurate normalization of real-time quantitative RT-PCR data by geometric averaging of multiple internal control genes. Genome Biol. *3*, RESEARCH0034.

Wang, F., Wang, J., Liu, D., and Su, Y. (2010). Normalizing genes for real-time polymerase chain reaction in epithelial and nonepithelial cells of mouse small intestine. Anal Biochem. *399*, 211–217.

Weidlich, I.E., and Filippov, I.V. (2016). Using the Gini coefficient to measure the chemical diversity of small-molecule libraries. J. Comput. Chem. *37*, 2091–2097.

Wilkinson, R., and Pickett, K. (2009). The Spirit Level: Why Equality Is Better for Everyone (Penguin Books).

Winter, G.E., Radic, B., Mayor-Ruiz, C., Blomen, V.A., Trefzer, C., Kandasamy, R.K., Huber, K.V.M., Gridling, M., Chen, D., Klampfl, T., et al. (2014). The solute carrier SLC35F2 enables YM155-mediated DNA damage toxicity. Nat. Chem. Biol. *10*, 768–773.

Zampieri, M., Ciccarone, F., Guastafierro, T., Bacalini, M.G., Calabrese, R., Moreno-Villanueva, M., Reale, A., Chevanne, M., Burkle, A., and Caiafa, P. (2010). Validation of suitable internal control genes for expression studies in aging. Mech. Ageing Dev. *131*, 89–95.

STAR★METHODS

KEY RESOURCES TABLE

REAGENT or RESOURCE	SOURCE	IDENTIFIER
**Software and Algorithms**	Gini coefficient	https://CRAN.R-project.org/package=ineq
**RESOURCE: Cell Atlas, cell line RNA-seq data**	Human Protein Atlas	https://www.proteinatlas.org/download/rna_celline.tsv.zip
**RESOURCE: Tissue Atlas, tissue RNA-seq data**	Human Protein Atlas	https://www.proteinatlas.org/download/rna_tissue.tsv.zip

CONTACT FOR REAGENT AND RESOURCE SHARING

Further information and requests for resources should be directed to and will be fulfilled by the Lead Contact, Douglas B. Kell (dbk@manchester.ac.uk).

METHOD DETAILS

The expression profile data are not new; the means by which they were obtained is described elsewhere (Thul et al., 2017; Uhlén et al., 2015). mRNA sequencing was performed on Illumina HiSeq2000 and 2,500 platforms (Illumina, San Diego, CA, USA) using the standard Illumina RNA-seq protocol with a read length of 2x100 bases. Transcript abundance estimation was performed using Kallisto (Bray et al., 2016) v0.42.4. For each gene, we report the abundance in 'Transcripts Per Million' (TPM) as the sum of the TPM values of all its protein-coding transcripts. For each cell line and tissue type, the average TPM values for replicate samples were used as abundance score. Thus each transcript level does represent an absolute value, but it is then normalised to the total expression in the particular sample. The data were extracted and extended in the form of Microsoft Excel sheets (Raw SLC and ABC data in Tables S1 and S2).

Most of the analyses are self-explanatory, but are noted below. As in many of our cheminformatics analyses (e.g. (O'Hagan and Kell, 2017a; O'Hagan et al., 2015)) we used the freely available KNIME software environment (Berthold et al., 2008; O'Hagan and Kell, 2015; O'Hagan et al., 2015) (http://knime.org/), with visualisation often provided via the Tibco Spotfire software (Perkin-Elmer Informatics).

Gini Index

The Gini Index was calculated using the **ineq** package (Achim Zeileis (2014). ineq: Measuring Inequality, Concentration, and Poverty. R package version 0.2-13. https://CRAN.R-project.org/package=ineq) in **R** (https://www.R-project.org/). These calculations were incorporated into KNIME via KNIME's R integration *R Snippet* node. The Rank Correlation used was Spearman's rho, using the KNIME *Rank Correlation node*.

Minimum and Maximum Expression Profiles

These and the other similar analyses were done using the functions contained in MS-Excel.

Immunohistochemistry

Immunohistochemical (IHC) images detailing protein expression patterns in 48 different normal tissues and 20 common cancer types are from the Human Protein Atlas database (www.proteinatlas.org). Tissue microarrays, immunostaining and image evaluation was performed as previously described (Uhlén et al., 2015). Briefly, 1mm duplicate cores were used for immunostaining using the following antibodies: HPA024575 for SLC22A12, HPA011885 for SLC6A18, HPA006539 for SLC2A14 (all from the Human Protein Atlas) and CAB037113 for PCBP1 (R1455 from Sigma-Aldrich). The immunostaining intensity and pattern was manually evaluated and scored by certified pathologists.

QUANTIFICATION AND STATISTICAL ANALYSIS

For each cell line and tissue type, the average TPM values for replicate samples were used as abundance score.

DATA AND SOFTWARE AVAILABILITY

The data on which we base our analyses are all available online at https://www.proteinatlas.org/about/download (and see Key Resources Table).

Cell Systems, Volume *6*

Supplemental Information

GeneGini: Assessment via the Gini Coefficient

of Reference “Housekeeping” Genes

and Diverse Human Transporter Expression Profiles

Steve O'Hagan, Marina Wright Muelas, Philip J. Day, Emma Lundberg, and Douglas B. Kell

SUPPLEMENTARY INFORMATION

The extra subsetted data that we give in the Supplementary Information are as follows:

Supplementary Table S1. Related to Fig 1. Expression profiles of the SLC transporters. Separate XL File: Supplementary Table S1 SLC_transporters_RNA_data_HPA.xlsx

Supplementary Table S2. Related to Fig 4. Expression profiles of the ABC transporters. Separate XL File: Supplementary Table S2 ABC_transporters_RNA_data_HPA.xlsx

Supplementary Table S3. Related to STAR Methods. A previously proposed set of useful reference genes, annotated here with their correct names and Uniprot IDs, together with their median expression levels and Gini indices in tissues as determined in this work.

Supplementary Table S4. Related to STAR Methods. Some genes that have previously been proposed as housekeeping or reference genes.

Supplementary Figures.

S1 (Relates to Fig 1.) Expression profiling of various SLC transporters in 59 tissues. Minimum and maximum expression levels of various SLCs in the 59 tissues considered (those with undetectable expression (i.e. <0.01 TPM, coded as zero) are not shown).

S2. (Relates to Fig 1.) Expression profiling of various SLC transporters in 59 tissues. The expression level of SLC35A4 is relatively homogeneous, with ¾ of all tissues within a factor two.

S3. (Relates to Fig 1.) Expression profiling of various SLC transporters in 59 tissues. The expression levels of SLC35F2 vary much more considerably, by a range of ~200 in these 59 tissue types.

S4. (Relates to Fig 1.) Expression profiling of various SLC transporters in 59 tissues. Expression profile of the transcripts for SLC22A4.

S5. (Relates to Fig 2.) Zoomed-in version of cluster 1 of Figure 2.

S6. (Relates to Fig 2.) Zoomed-in version of cluster 2 of Figure 2.

S7. (Relates to Fig 2.) Zoomed-in version of cluster 3 of Figure 2.

S8. (Relates to Fig 2.) Zoomed-in version of cluster 4 of Figure 2.

Gene name	Protein name	Uniprot ID	Gini index in tissues	Median expression level (TPM)
C1orf43	Chromosome 1 open reading frame 43	Q9BWL3	0.204	137
CHMP2A	Charged multivesicular body protein 2A	O43633	0.141	126
EMC7	ER membrane protein complex subunit 7	Q9NPA0	0.210	69
GPI	Glucose-6-phosphate isomerase	P06744	0.259	137
PSMB2	Proteome subunit beta type 2	P49721	0.186	32
PSMB4	Proteome subunit beta type 4	P28070	0.200	209
RAB7A	RAS-related protein 7A	P51149	0.171	167
REEP5	Receptor expression-enhancing protein 5	Q00765	0.315	65
SNRPD3	Small nuclear ribonucleoprotein Sm D3	P62318	0.192	55
VCP	Transitional endoplasmic reticulum ATPase (originally valosin containing protein)	P55072	0.198	48
VPS29	Vacuolar protein sorting associated protein 29	Q9UBQ0	0.146	74

Supplementary Table S3. A previously proposed set of useful reference genes, annotated here with their correct names and Uniprot IDs, together with their median expression levels and Gini indices in tissues as determined in this work.

Gene	Protein	Uniprot ID	Gini index
GAPDH	Glyceraldehyde 3-phosphate dehydrogenase	P04406	0.344
LDHA	Lactate dehydrogenase subunit A	P00338	0.32
SDHA	Succinate dehydrogenase subunit A	P31040	0.308
HRPT1	Hypoxanthine phosphoribosyl transferase 1	P00492	0.277
HBS1L	*HBS1*-like protein	Q9Y450	0.184
OAZ1	Ornithine decarboxylase antizyme 1	P54368	0.202
PPIA1	Peptidyl-prolyl cis-trans isomerase	P62937	0.24
AHSP	Alpha-haemoglobin stabilising protein	Q9NZD4	0.97
B2M	β_2-microglobulin	P61769	0.349
ACTB	β-actin	P60709	0.291
HMBS	Porphobilinogen deaminase	P08397	0.303
UBC	Polyubiquitin C	P0CG48	0.183
POLR2F	DNA-directed RNA polymerases I, II, and III subunit RPABC2	P61218	0.235
GUSB	β-glucuronidase	P08236	0.25
TBP	TATA-box binding protein	P20226	0.22
YWHAZ	14-3-3 protein zeta/delta	P63104	0.255

Supplementary Table S4. Some genes that have previously been proposed as housekeeping or reference genes.

S1

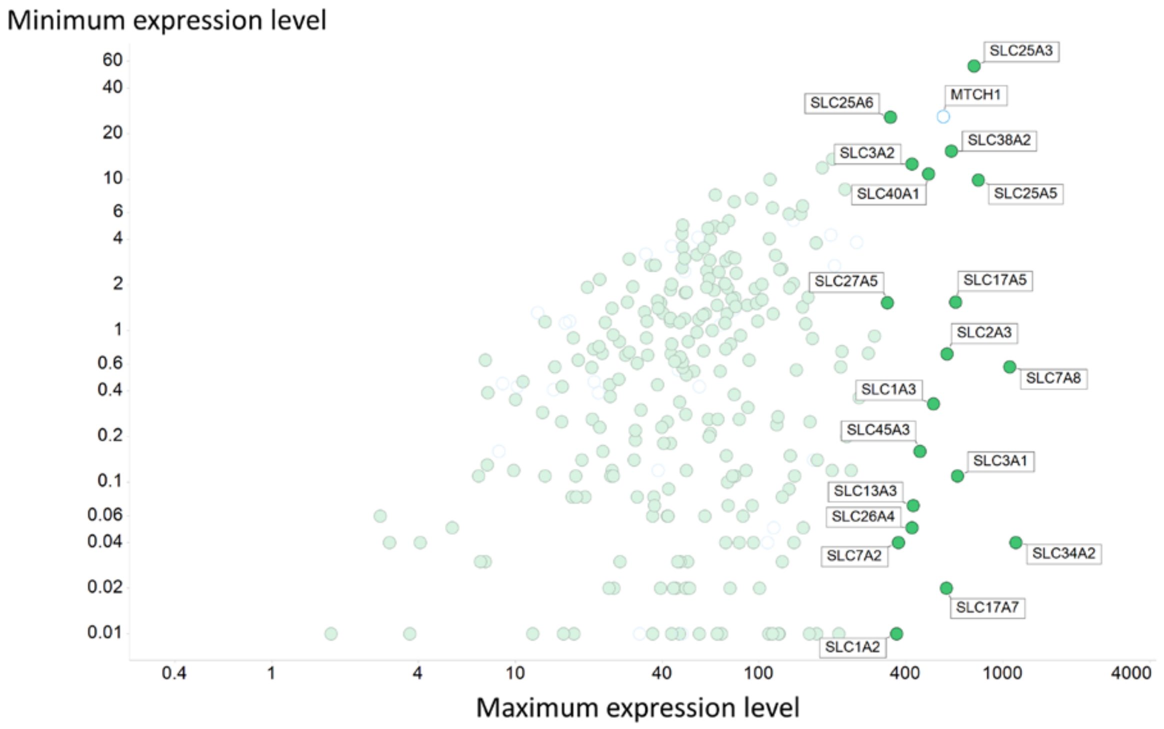

Supplementary Fig S1 (Relates to Fig 1.) Expression profiling of various SLC transporters in 59 tissues. Minimum and maximum expression levels of various SLCs in the 59 tissues considered (those with undetectable expression (i.e. <0.01 TPM, coded as zero) are not shown).

S2

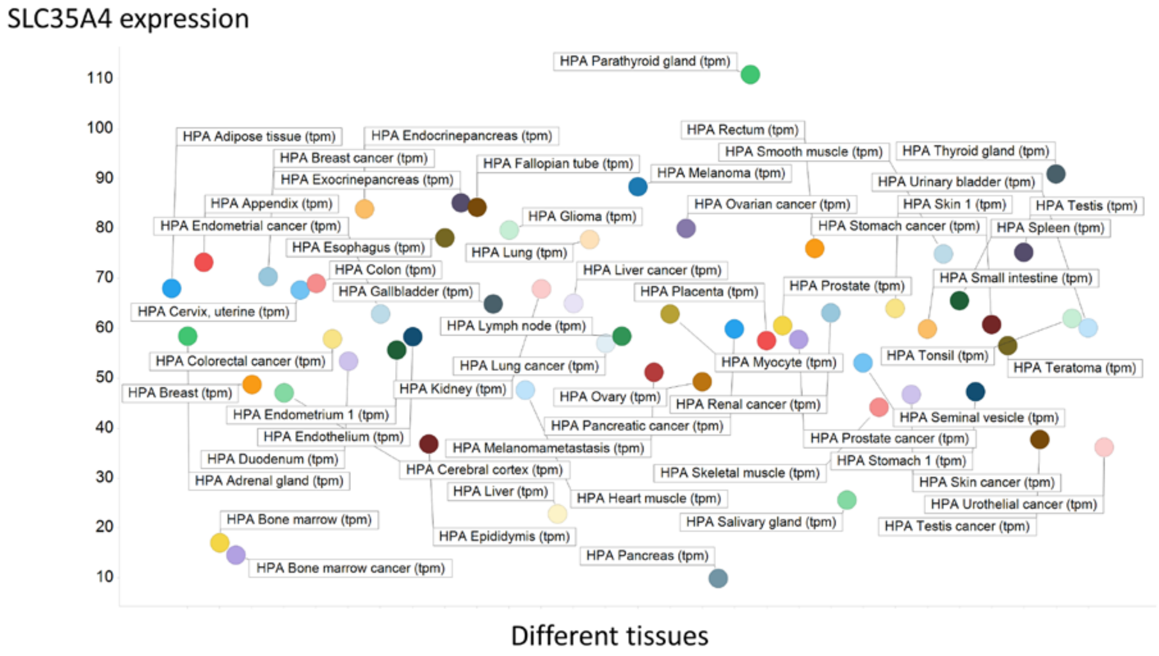

Supplementary Fig S2. (Relates to Fig 1.) Expression profiling of various SLC transporters in 59 tissues. The expression level of SLC35A4 is relatively homogeneous, with ¾ of all tissues within a factor two.

S3

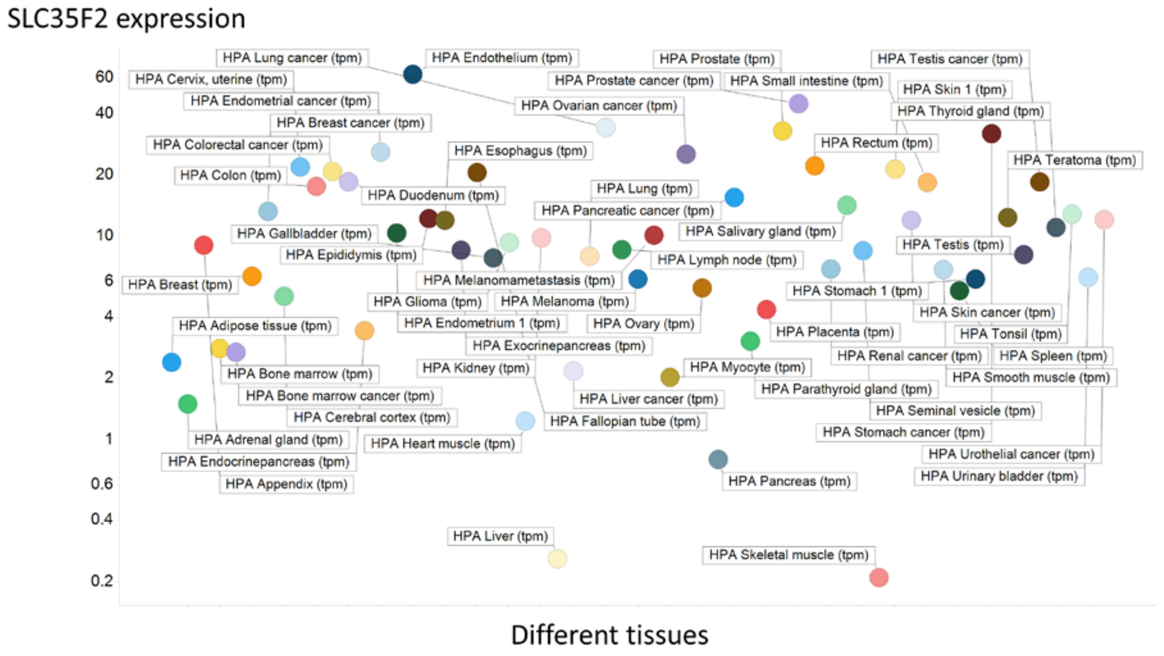

Supplementary Fig S3. (Relates to Fig 1.) Expression profiling of various SLC transporters in 59 tissues. The expression levels of SLC35F2 vary much more considerably, by a range of ~200 in these 59 tissue types.

S4

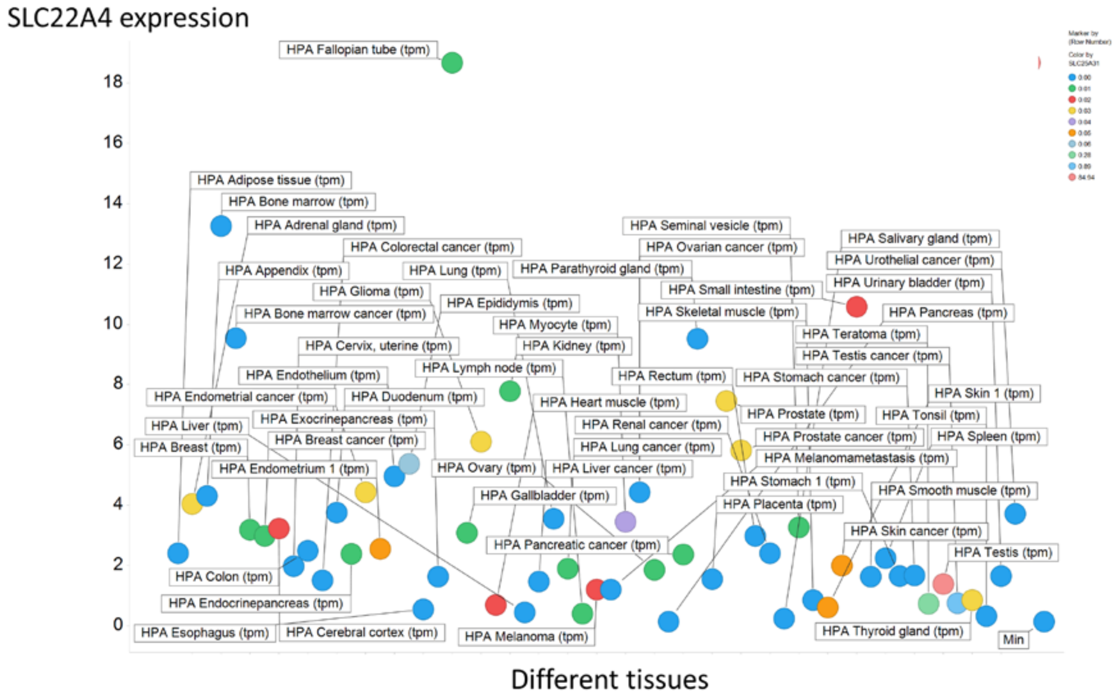

Supplementary Fig S4. (Relates to Fig 1.) Expression profiling of various SLC transporters in 59 tissues. Expression profile of the transcripts for SLC22A4.

S5

Cluster 1

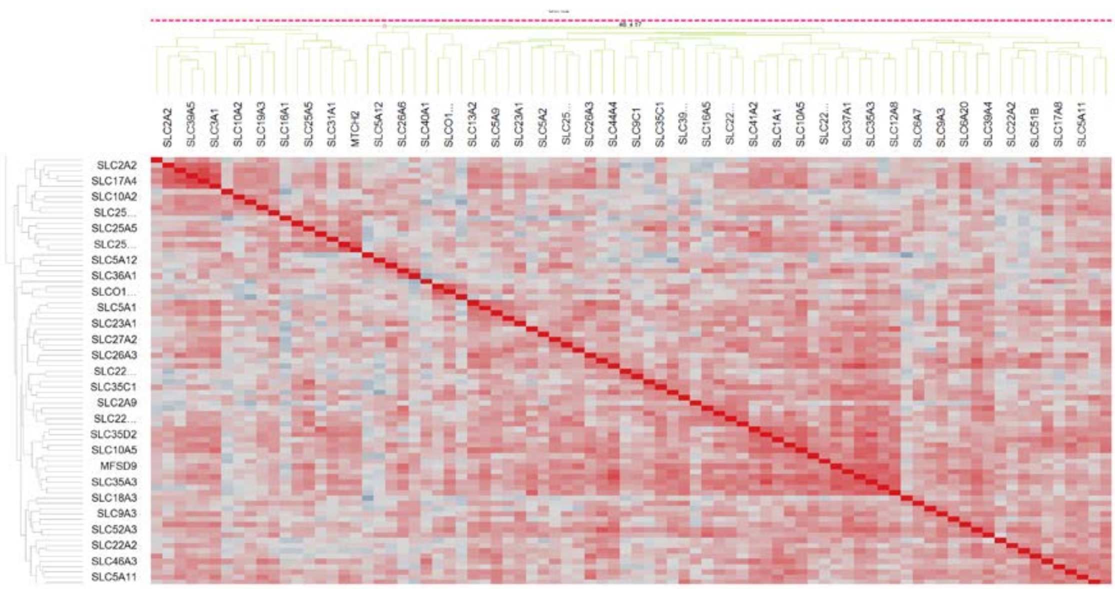

Supplementary Fig S5. (Relates to Fig 2.) Zoomed-in version of cluster 1 of Figure 2.

S6

Cluster 2

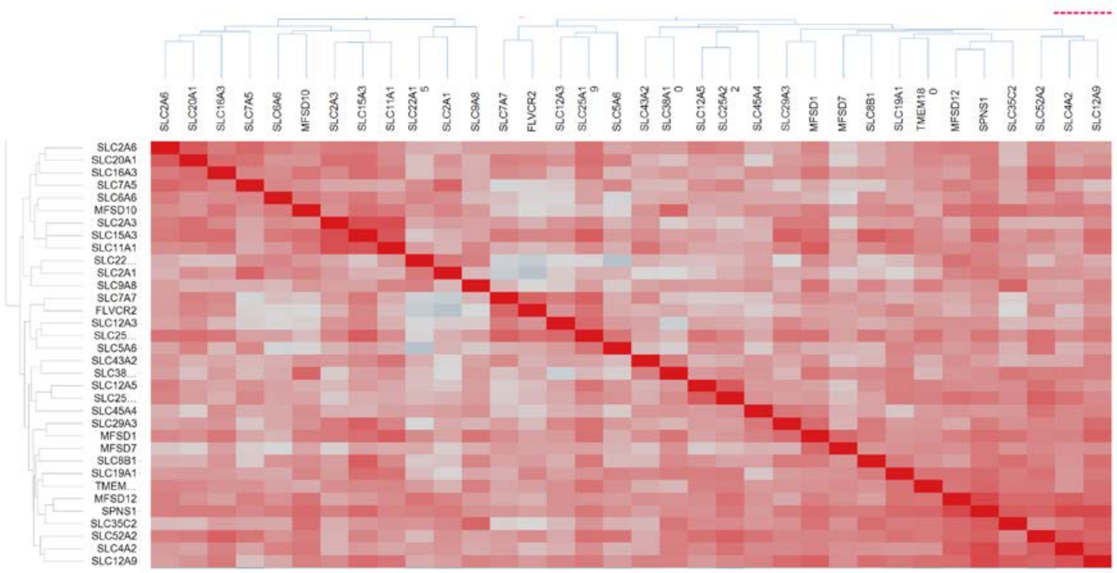

Supplementary Fig S6. (Relates to Fig 2.) Zoomed-in versions cluster2 of Figure 2.

S7

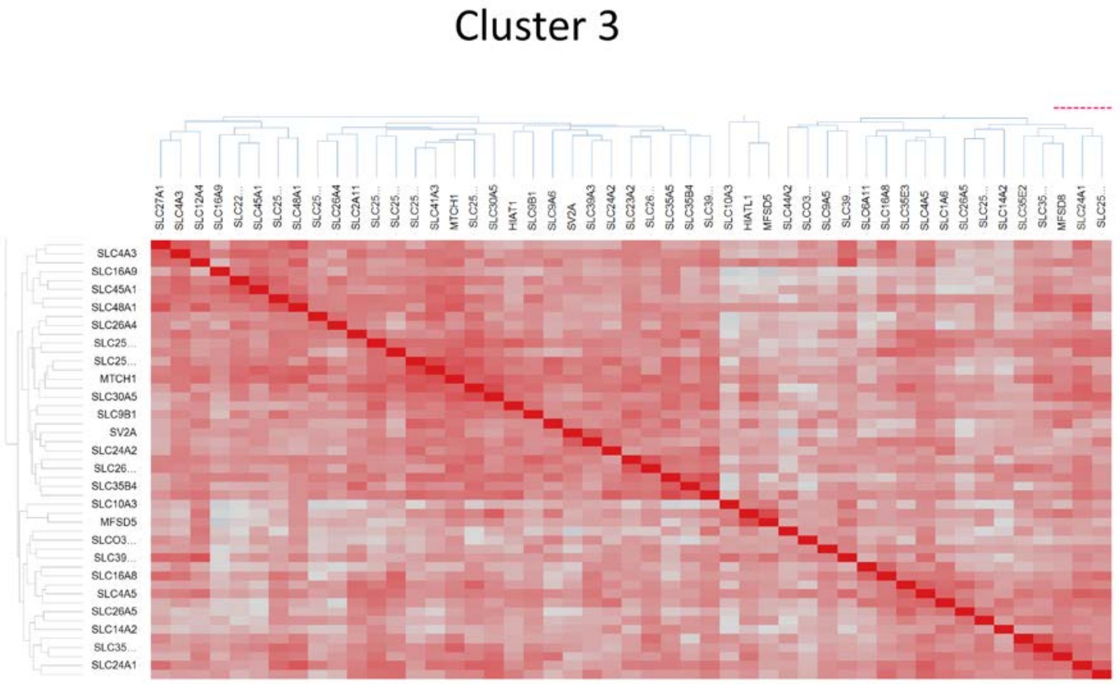

Supplementary Fig S7. (Relates to Fig 2.) Zoomed-in version cluster 3 of Figure 2.

S8

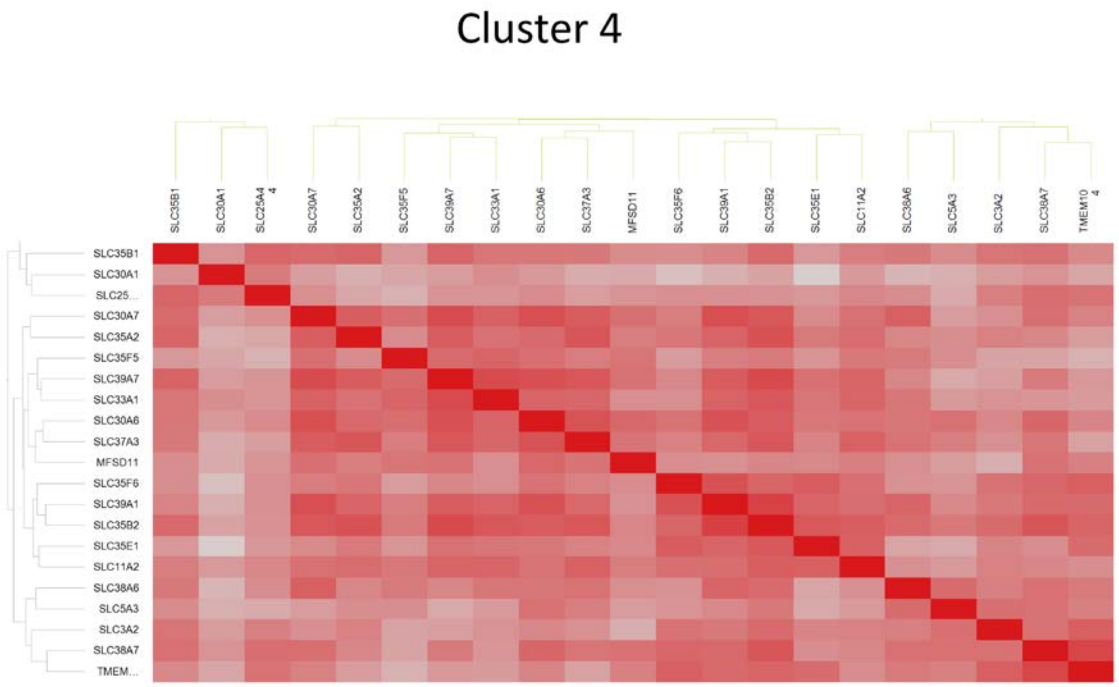

Supplementary Fig S8. (Relates to Fig 2.) Zoomed-in version of cluster 4 of Figure 2.
